# microRNA-9 Suppresses the Proliferation, Invasion and Metastasis of Gastric Cancer Cells through Targeting Cyclin D1 and Ets1

**DOI:** 10.1371/journal.pone.0055719

**Published:** 2013-01-31

**Authors:** Liduan Zheng, Teng Qi, Dehua Yang, Meng Qi, Dan Li, Xuan Xiang, Kai Huang, Qiangsong Tong

**Affiliations:** 1 Department of Pathology, Union Hospital of Tongji Medical College, Huazhong University of Science and Technology, Wuhan, Hubei Province, China; 2 Clinical Center of Human Genomic Research, Union Hospital of Tongji Medical College, Huazhong University of Science and Technology, Wuhan, Hubei Province, China; 3 Department of Surgery, Union Hospital of Tongji Medical College, Huazhong University of Science and Technology, Wuhan, Hubei Province, China; 4 Department of Cardiology, Union Hospital of Tongji Medical College, Huazhong University of Science and Technology, Wuhan, Hubei Province, China; Pontificia Universidad Catolica de Chile, Chile

## Abstract

Recent evidence shows that altered microRNA-9 (miR-9) expression is implicated in the progression of gastric cancer. However, the exact roles and underlying mechanisms of miR-9 in the proliferation, invasion and metastasis of gastric cancer still remain unknown. In this study, miR-9 was found to be down-regulated and inversely correlated with the expression of cyclin D1 and v-ets erythroblastosis virus E26 oncogene homolog 1 (Ets1) in gastric cancer tissues and cell lines. Bioinformatics analysis revealed the putative miR-9 binding sites in the 3′-untranslated regions (3′-UTR) of cyclin D1 and Ets1 mRNA. Ectopic expression or knockdown of miR-9 resulted in responsively altered expression of cyclin D1, Ets1 and their downstream targets phosphorylated retinoblastoma and matrix metalloproteinase 9 in cultured gastric cancer cell lines SGC-7901 and AGS. In the luciferase reporter system, miR-9 directly targeted the 3′-UTR of cyclin D1 and Ets1, and these effects were abolished by mutating the miR-9 binding sites. Over-expression of miR-9 suppressed the proliferation, invasion, and metastasis of SGC-7901 and AGS cells *in vitro* and *in vivo*. Restoration of miR-9-mediated down-regulation of cyclin D1 and Ets1 by transient transfection, rescued the cancer cells from decrease in proliferation, migration and invasion. Furthermore, anti-miR-9 inhibitor promoted the proliferation, migration and invasion of gastric cancer cells, while knocking down of cyclin D1 or Ets1 partially phenocopied the effects of miR-9 over-expression. These data indicate that miR-9 suppresses the expression of cyclin D1 and Ets1 via the binding sites in their 3′-UTR, thus inhibiting the proliferation, invasion and metastasis of gastric cancer.

## Introduction

Gastric cancer is the fourth most common cancer in the world [1]. In spite of the improvement in surgical and multimodal therapy, the prognosis of advanced gastric cancer still remains poor due to the recurrence, invasion and metastasis, with a 5-year survival rate below 30% [1]. A better knowledge of the mechanisms underlying tumor progression is warranted to discover novel paradigms for the diagnosis and treatment of gastric cancer [2]. MicroRNAs (miRNAs), a recently identified category of small and highly conserved noncoding RNAs, can participate in the post-transcriptional regulation of gene expression through partial complementary binding with the 3′ untranslated regions (3′-UTRs) of target mRNA, resulting in translational repression or mRNA degradation [3]. Emerging evidence shows that miRNAs are involved in the biological processes related to apoptosis, proliferation, differentiation, invasion and metastasis, while deregulation of which is crucial to cancer initiation and progression [3]. It is currently urgent to investigate the roles of miRNAs and their target genes in tumor progression by experimental models.

In human gastric cancer, a number of miRNAs have been reported to be aberrantly over-expressed or down-regulated during the progression of gastric cancer, including miR-21 [4], miR-15b [5], miR-16 [5], and miR-101 [6]. These miRNAs play oncogenic or tumor-suppressive roles in the regulation of cell growth, migration and invasion by repressing their target genes. For example, oncogenic miR-21 is aberrantly over-expressed in gastric cancer, and enhances the proliferation and invasiveness of gastric cancer cells by targeting reversion-inducing cysteine-rich protein with Kazal motifs [4]. Meanwhile, miR-15b and miR-16 are down-regulated in gastric cancer, and contribute to multidrug resistance of gastric cancer cells by modulation of apoptosis via targeting B-cell leukemia/lymphoma 2 [5]. miR-101 is down-regulated in gastric cancer tissues and cell lines, while ectopic expression of miR-101 significantly inhibits the proliferation, migration and invasion of gastric cancer cells by targeting enhancer of zeste homolog 2, cytochrome c oxidase subunit II, and myeloid cell leukemia sequence 1 [6]. Therefore, it has been a focus to further explore the expression and function of miRNA in the tumor biology of gastric cancer.

MicroRNA-9 (miR-9) is first identified as one of the crucial regulators for the development, physiology and pathology of nervous system in several organisms, including *Drosophila*, zebrafish, and mammals [7]–[9]. A series of subsequent studies have shown that altered miR-9 expression is associated with the development and progression of cancers [10]–[14]. Previous studies indicate that miR-9 is significantly down-regulated in gastric cancer, implying its potential roles in tumor progression [15]–[18]. It is also indicated that miR-9 can modulate the proliferation of gastric cancer cells via targeting the caudal type homeobox 2 (CDX2) [19] and NF-kappa B (NF-κB) [16]. However, the exact function and underlying mechanisms of miR-9 in the progression of gastric cancer still warrant further investigation. In this study, we demonstrate, for the first time, that miR-9 directly targets cyclin D1 and v-ets erythroblastosis virus E26 oncogene homolog 1 (Ets1), and suppresses the proliferation, invasion and metastasis of gastric cancer cells *in vitro* and *in vivo*.

## Results

### miR-9 was down-regulated and inversely correlated with the expression of cyclin D1 and Ets1 in gastric cancer tissues and cell lines

**Figure 1:**
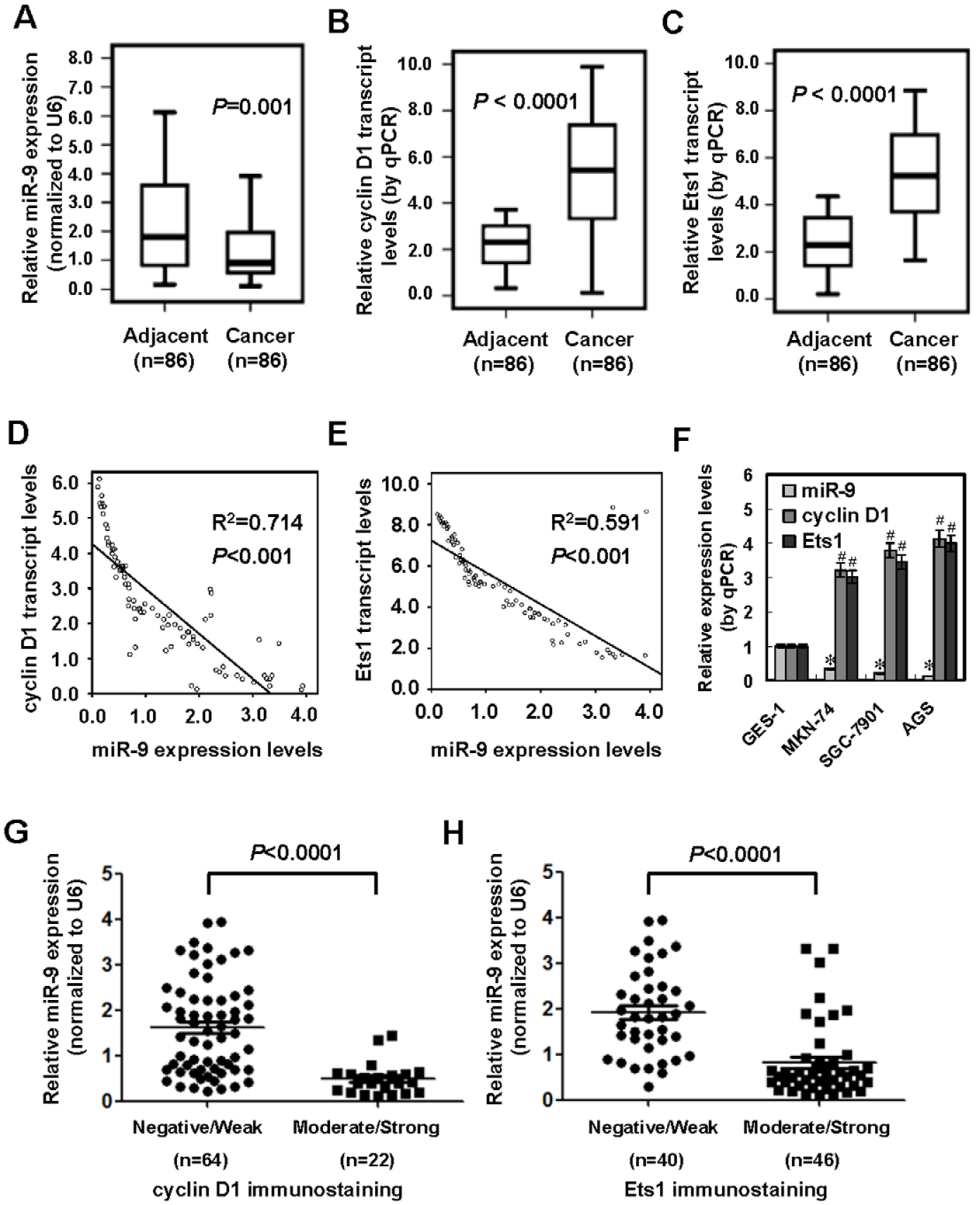
miR-9 was down-regulated and inversely correlated with the expression of cyclin D1 and Ets1 in gastric cancer tissues and cell lines. **A**, **B** and **C**, real-time quantitative RT-PCR indicated that when compared to adjacent non-neoplastic mucosa (n = 86), down-regulation of miR-9 and higher transcript levels of cyclin D1 and Ets1 were detected in gastric cancer tissues (n = 86). **D** and **E**, there was an inverse correlation between miR-9 expression and transcript levels of cyclin D1 or Ets1 in gastric cancer tissues. **F**, lower miR-9 expression and higher cyclin D1 and Ets1 transcript levels were observed in gastric cancer cell lines (MKN-74, SGC-7901 and AGS), when compared to those in normal gastric epithelial GES-1 cells. **G** and **H**, lower miR-9 expression was observed in gastric cancer tissues with higher immunostaining of cyclin D1 or Ets1. The symbols (* and #) indicate a significant decrease and a significant increase from GES-1, respectively.

To investigate the miR-9 expression in gastric cancer, gastric cancer tissues and adjacent non-neoplastic mucosa were collected from 86 primary cases. Real-time quantitative reverse transcription PCR (RT-PCR) revealed that miR-9 was down-regulated in gastric cancer tissues than that in adjacent non-neoplastic mucosa (*P* = 0.001, Fig. 1A). The miR-9 expression was significantly lower in gastric cancer cases with deeper gastric wall invasion (*P*<0.001), lymph node metastasis (*P*<0.001), distant metastasis (*P* = 0.022), and advanced TNM stage (*P* = 0.01) (Table S1). In contrast, higher cyclin D1 and Ets1 transcript levels were detected in gastric cancer tissues (*P*<0.0001 and *P*<0.0001, respectively, Fig. 1B and Fig. 1C). There was an inverse correlation between miR-9 expression and cyclin D1 transcript levels in gastric cancer tissues (*P*<0.001, Fig. 1D). In addition, an inverse correlation between miR-9 expression and Ets1 transcript levels was also noted in these cancer tissues (*P*<0.001, Fig. 1E). Lower miR-9 expression and higher transcript levels of cyclin D1 and Ets1 were observed in gastric cancer cell lines than those in normal gastric epithelial GES-1 cells [20] (Fig. 1F). Immunohistochemical staining was performed to observe the expression of cyclin D1 and Ets1 in these cancer specimens (Fig. S1). Nuclear cyclin D1 was noted in 26/86 (30.2%) cases, while nuclear and cytoplasm staining of Ets1 was observed in 58/86 (67.4%) cases (Table S1). The immunoreactivity of cyclin D1 and Ets1 was significantly higher in gastric cancer cases with deeper gastric wall invasion (*P*<0.001 and *P* = 0.001), lymph node metastasis (*P*<0.001 and *P*<0.001), distant metastasis (*P*<0.001 and *P*<0.001), and advanced TNM stage (*P*<0.001 and *P* = 0.004) (Table S1). Lower miR-9 expression was observed in gastric cancer tissues with higher immunostaining of cyclin D1 (*P*<0.0001, Fig. 1G) or Ets1 (*P*<0.0001, Fig. 1H). These results indicated that miR-9 was down-regulated and inversely correlated with expression of cyclin D1 and Ets1 in gastric cancer tissues and cell lines.

### miR-9 down-regulated the expression of cyclin D1 and Ets1 through post-transcriptional repression

**Figure 2:**
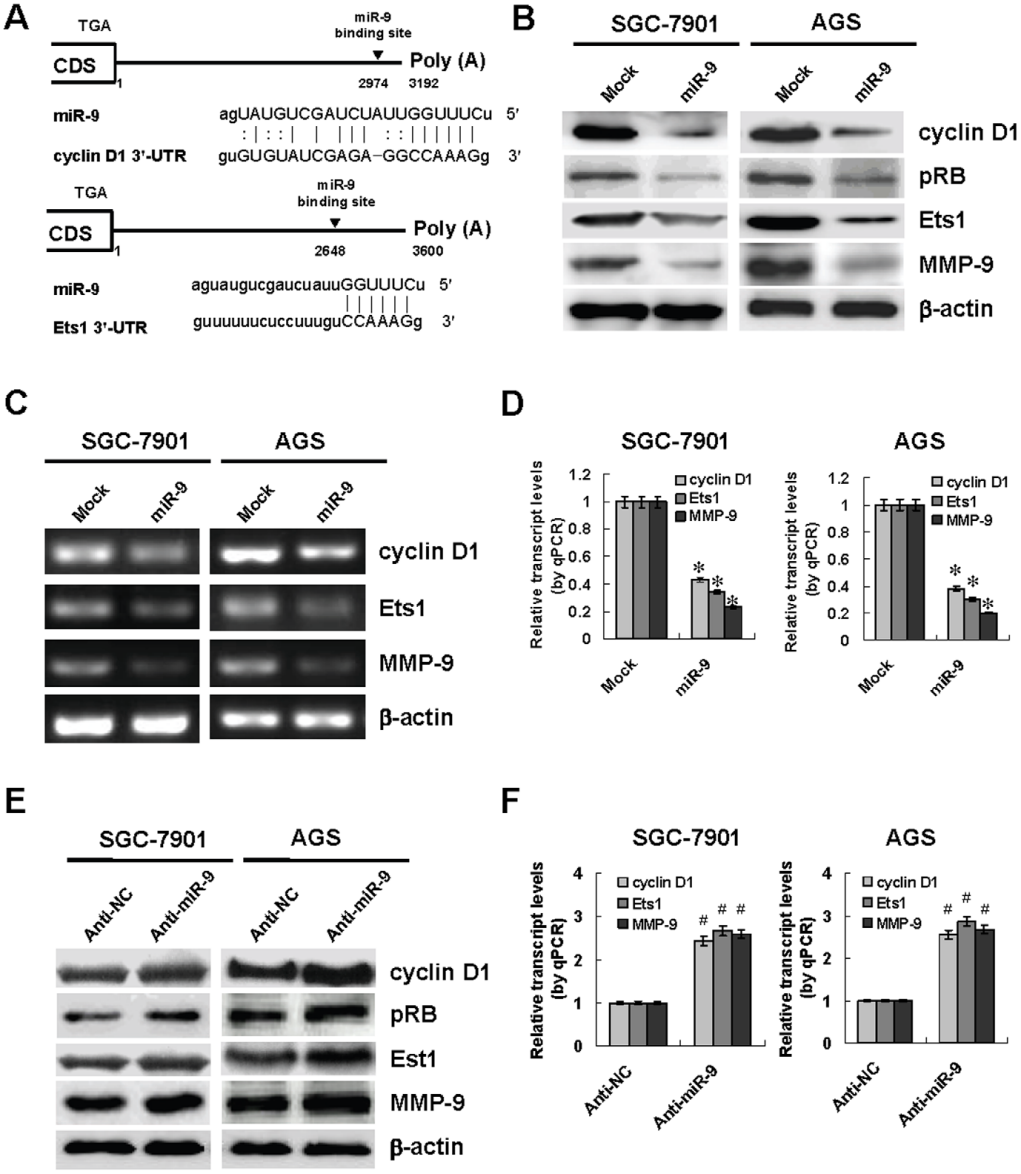
miR-9 suppressed the expression of cyclin D1 and Ets1 through post- transcriptional repression in gastric cancer cells. **A**, scheme of the potential binding sites of miR-9 in the 3′-UTR of cyclin D1 and Ets1, locating at bases 2974–2995 and 2648–2670, respectively. **B**, stable transfection of miR-9 precursor resulted in decreased protein levels of cyclin D1, Ets1 and their downstream targets pRB and MMP-9 in gastric cancer SGC-7901 and AGS cells than those transfected with negative control vector (mock). **C** and **D**, the transcript levels of cyclin D1, Ets1 and MMP-9 were decreased in miR-9 precursor-transfected SGC-7901 and AGS cells, when compared to those in mock cells. **E**, transfection of anti-miR-9 inhibitor (100 nmol/L) resulted in increased protein levels of cyclin D1, Ets1 and their downstream targets pRB and MMP-9 in SGC-7901 and AGS cells than those transfected with negative control inhibitor (anti-NC, 100 nmol/L). **F**, the transcript levels of cyclin D1, Ets1, and MMP-9 were increased in SGC-7901 and AGS cells transfected with anti-miR-9 inhibitor (100 nmol/L), when compared to those transfected with anti-NC (100 nmol/L). The symbols (* and #) indicate a significant decrease and a significant increase from mock or anti-NC, respectively.

To investigate the hypothesis that miR-9 may influence the expression of cyclin D1 and Ets1 in gastric cancer, computational prediction was performed by miRNA databases. Potential binding sites of miR-9 with high complementarity were noted at bases 2974–2995 of the cyclin D1 3′-UTR and 2648–2670 of the Ets1 3′-UTR (Fig. 2A). To investigate the direct effects of miR-9 on the expression of cyclin D1 and Ets1 in gastric cancer cells, we performed the miRNA over-expression experiments. Stable transfection of miR-9 precursor into SGC-7901 and AGS cells resulted in increase of miR-9 levels (Fig. S2). Western blot, RT-PCR and real-time quantitative RT-PCR demonstrated that over-expression of miR-9 resulted in decreased protein and transcriptional levels of cyclin D1 and Ets1 in gastric cancer cells than those transfected with negative control vector (mock) (Fig. 2B, Fig. 2C, and Fig. 2D). In addition, the levels of phosphorylated retinoblastoma (pRB, a downstream effector of cyclin D1) [21] and matrix metalloproteinase 9 (MMP-9, a downstream gene of Ets1) [22] were decreased in miR-9 over-expressing cancer cells (Fig. 2B, Fig. 2C, and Fig. 2D). To further examine the suppressive role of miR-9 in the expression of cyclin D1 and Ets1, we performed the miR-9 knockdown experiments by transfection of anti-miR-9 or negative control (anti-NC) inhibitors into GES-1, SGC-7901 and AGS cells. Transfection of anti-miR-9 inhibitor obviously decreased the endogenous miR-9 expression (Fig. S3A), and upregulated the protein levels of cyclin D1, pRB, Ets1 and MMP-9 than those transfected with anti-NC (Fig. 2E and Fig. S4A). Real-time quantitative RT-PCR analyses showed the enhanced transcript levels of cyclin D1, Ets1 and MMP-9 in cultured cells transfected with anti-miR-9 inhibitor, when compared to those transfected with anti-NC (Fig. 2F and Fig. S4B). Overall, these results demonstrated that miR-9 considerably inhibited the expression of cyclin D1 and Ets1 through post-transcriptional repression.

### miR-9 directly targeted cyclin D1 and Ets1 in gastric cancer cells

**Figure 3:**
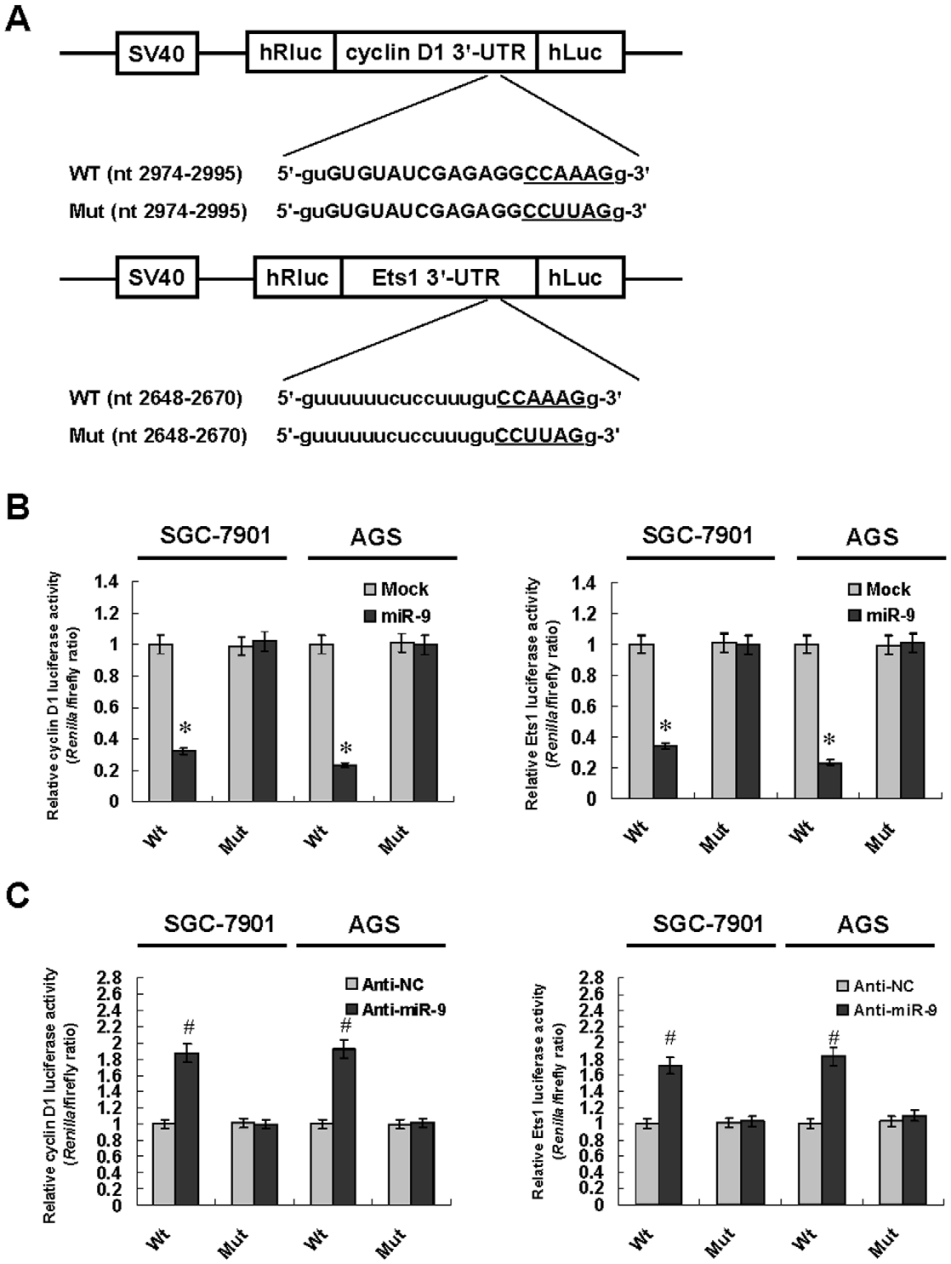
miR-9 directly interacted with the putative binding sites in the 3′-UTR of cyclin D1 and Ets1. **A**, scheme and sequence of the intact miR-9 binding site (Wt) and its mutation (Mut) within the luciferase reporter vectors. **B**, stable transfection of miR-9 precursor into SGC-7901 and AGS cells resulted in decreased luciferase activities of 3′-UTR reporter of cyclin D1 and Ets1 than those transfected with negative control vector (mock), which were abolished by mutation in the putative miR-9 binding sites. **C**, transfection of anti-miR-9 inhibitor (100 nmol/L) into SGC-7901 and AGS cells increased the luciferase activities than those transfected with anti-NC (100 nmol/L), while mutation of miR-9 recognition site abolished these effects. The symbols (* and #) indicate a significant decrease and a significant increase from mock or anti-NC, respectively.

To determine whether miR-9 could repress the expression of cyclin D1 and Ets1 by targeting its binding sites in the 3′-UTR, the PCR products containing intact target sites or mutation of miR-9 seed recognition sequences (Fig. 3A) were inserted into the luciferase reporter vector. The plasmids were transfected into gastric cancer cells stably transfected with negative control vector (mock) or miR-9 precursor. The *Renilla* luciferase activities normalized to those of firefly were significantly reduced in SGC-7901 and AGS cells stably transfected with miR-9 precursor (Fig. 3B), and these effects were abolished by mutating the putative miR-9 binding sites within the 3′-UTR of cyclin D1 and Ets1 (Fig. 3B). Moreover, knockdown of miR-9 with anti-miR-9 inhibitor increased the luciferase activities in GES-1, SGC-7901 and AGS cells (Fig. 3C and Fig. S4C), while mutation of miR-9 recognition site abolished these effects (Fig. 3C and Fig. S4C). These results indicated that miR-9 directly and specifically interacted with the target sites in the 3′-UTR of cyclin D1 and Ets1.

### miR-9 suppressed the *in vitro* proliferation of gastric cancer cells through targeting cyclin D1

**Figure 4:**
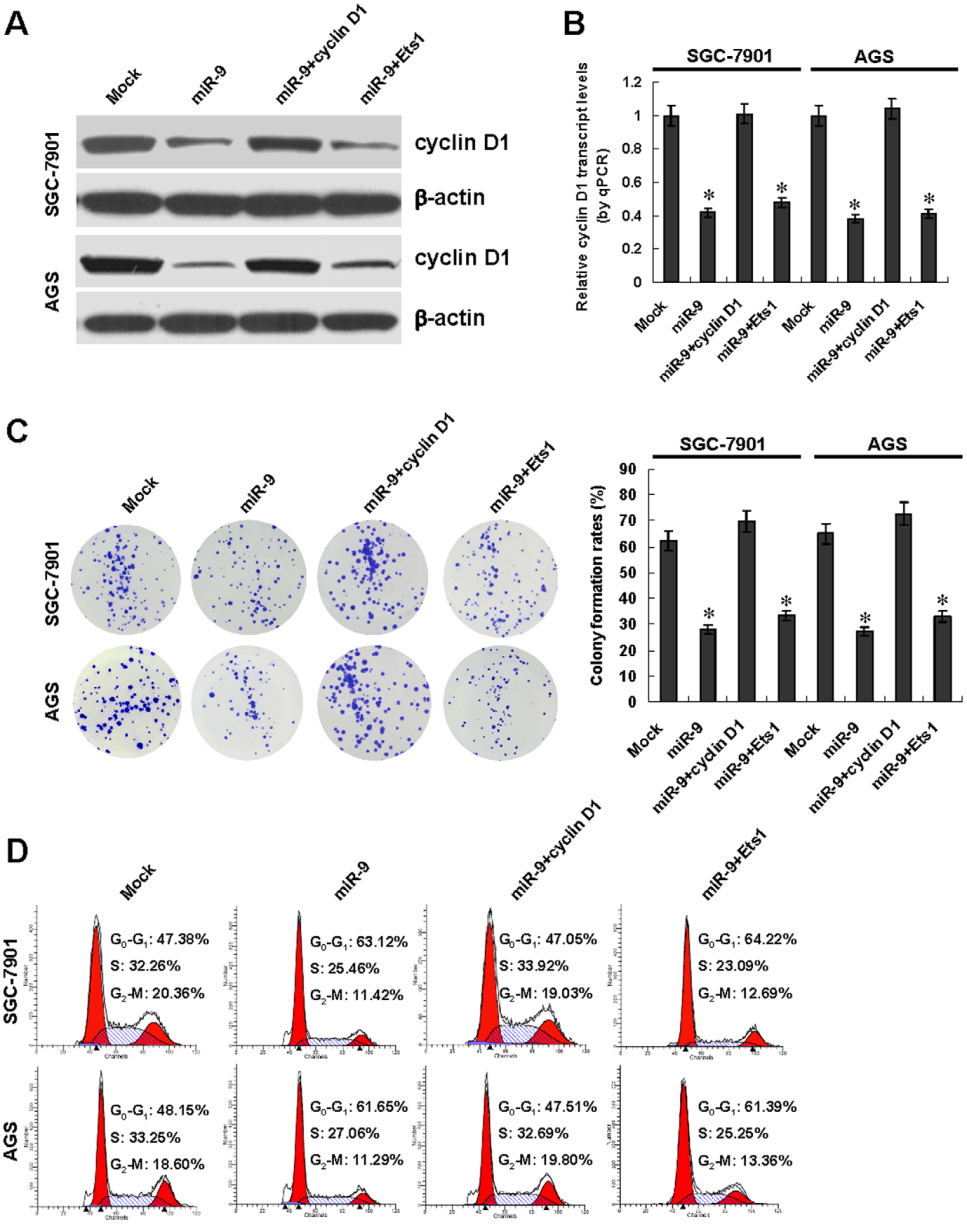
Ectopic expression of miR-9 abolished the *in vitro* proliferation of gastric cancer cells through targeting cyclin D1. **A** and **B**, western blot and real-time quantitative RT-PCR indicated that transfection of cyclin D1, but not of Ets1, restored the down-regulation of cyclin D1 induced by miR-9 over-expression in gastric cancer SGC-7901 and AGS cells, when compared to those transfected with negative control vector (mock). **C**, in colony formation assay, miR-9 over-expression attenuated the growth of SGC-7901 and AGS cells than that of mock cells, which was rescued by transfection of cyclin D1, but not of Ets1. **D**, flow cytometry indicated that miR-9 over-expression induced the cell cycle arrest at G_0_/G_1_ phase in SGC-7901 and AGS cells than in mock cells, which was rescued by transfection of cyclin D1, but not of Ets1. The symbol (*) indicates a significant decrease from mock.

Since above evidence showed that miR-9 inhibited the cyclin D1 expression, and combining the facts that cyclin D1 plays an critical role in the cell cycle progression and proliferation of cancer cells [21], we further investigated the effects of miR-9 over-expression and target gene restoration on cultured gastric cancer cells. Western blot and real-time quantitative RT-PCR indicated that transfection of cyclin D1, but not of Ets1, rescued the miR-9-induced down-regulation of cyclin D1 (Fig. 4A and Fig. 4B). In colony formation assay, miR-9 over-expression attenuated the growth of SGC-7901 and AGS cells, when compared to those transfected with negative control vector (mock) (Fig. 4C). Flow cytometry indicated that miR-9 over-expression induced cell cycle arrest at G_0_/G_1_ phase in SGC-7901 and AGS cells (Fig. 4D). In addition, transfection of cyclin D1, but not of Ets1, into SGC-7901 and AGS cells restored the proliferation inhibition and cell cycle arrest induced by over-expression of miR-9 (Fig. 4C and Fig. 4D). On the other hand, we examined the effects of miR-9 knockdown on GES-1, SGC-7901 and AGS cells. Introduction of anti-miR-9 inhibitor into these cells resulted in enhanced abilities in proliferation (Fig. S3B) and cell cycle progression (Fig. S3C). These results indicated that miR-9 remarkably suppressed the *in vitro* proliferation and cell cycle progression of gastric cancer cells through targeting cyclin D1.

### miR-9 attenuated the *in vitro* migration and invasion of gastric cancer cells through targeting Ets1

**Figure 5:**
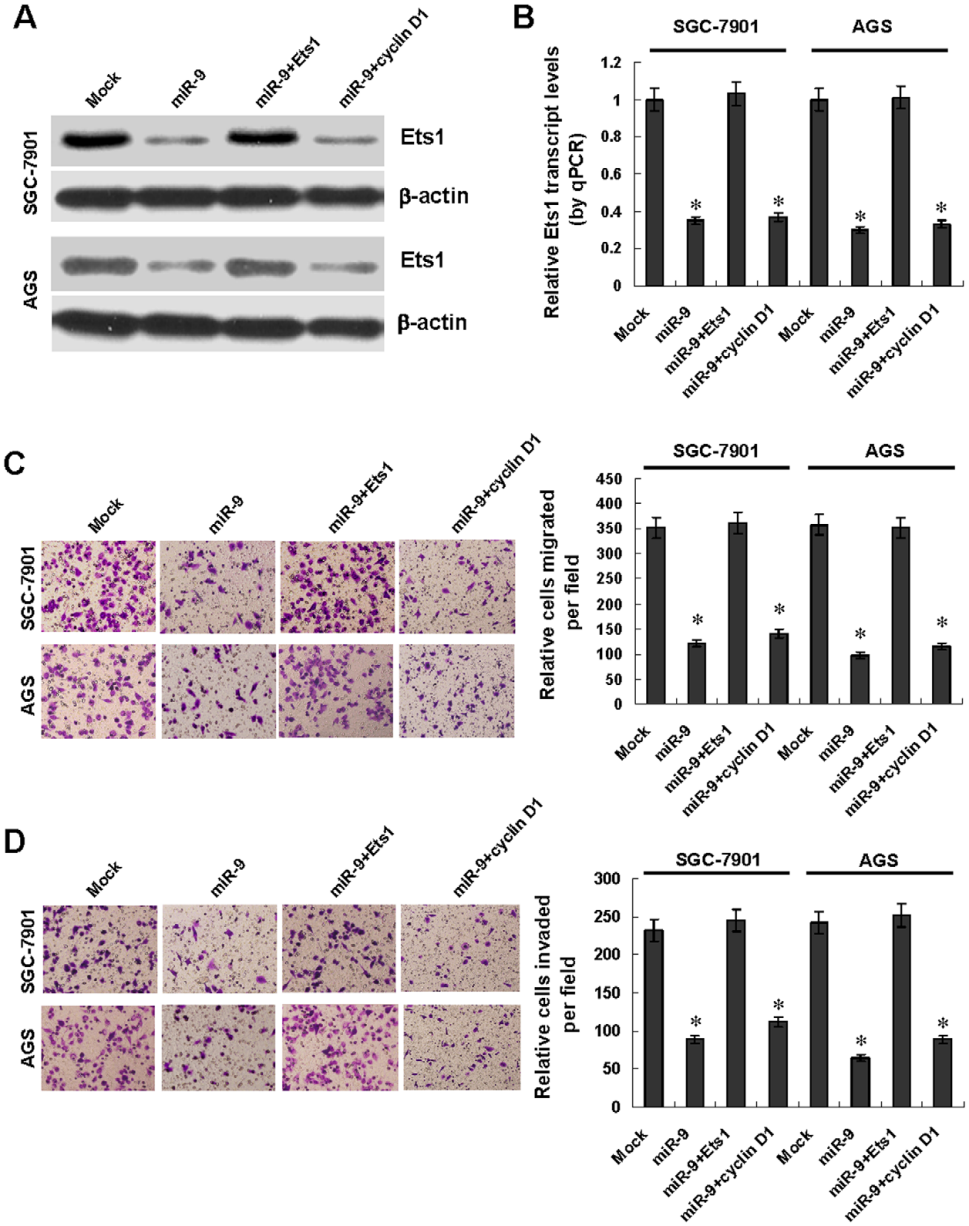
Over-expression of miR-9 attenuated the *in vitro* migration and invasion of gastric cancer cells through targeting Ets1. **A** and **B**, western blot and real-time quantitative RT-PCR indicated that transfection of Ets1, but not of cyclin D1, restored the down-regulation of Ets1 induced by miR-9 over-expression in gastric cancer SGC-7901 and AGS cells, when compared to those transfected with negative control vector (mock). **C**, in transwell migration assay, gastric cancer cells stably transfected with miR-9 precursor presented an impaired migration capacity than that in mock cells, which was rescued by transfection of Ets1, but not of cyclin D1. **D**, in matrigel invasion assay, miR-9 over-expression attenuated the invasion capabilities of SGC-7901 and AGS cells than those of mock cells, which was rescued by transfection of Ets1, but not of cyclin D1. The symbol (*) indicates a significant decrease from mock.

Since previous studies indicate the important roles of Ets1 in the invasion and metastasis of cancer cells [23], [24], we further investigated the effects of miR-9 over-expression and target gene restoration on the migration and invasion of gastric cancer SGC-7901 and AGS cells. Western blot and real-time quantitative RT-PCR indicated that transfection of Ets1, but not of cyclin D1, rescued the miR-9-induced down-regulation of Ets1 (Fig. 5A and Fig. 5B). In transwell migration assay, gastric cancer cells stably transfected with miR-9 precursor presented an impaired migration capacity than those transfected with negative control vector (mock) (Fig. 5C). In matrigel invasion assay, miR-9 over-expression attenuated the invasiveness of SGC-7901 and AGS cells (Fig. 5D). In addition, transfection of Ets1, but not of cyclin D1, into SGC-7901 and AGS cell lines restored the miR-9-meditaed inhibition on the migration and invasion (Fig. 5C and Fig. 5D). Furthermore, we examined the effects of miR-9 knockdown on GES-1, SGC-7901 and AGS cells. Transfection of anti-miR-9 inhibitor resulted in enhanced migration and invasion of these cells (Fig. S3D and Fig. S3E). These results indicated that miR-9 remarkably attenuated the *in vitro* migration and invasion of gastric cancer cells through targeting Ets1.

### miR-9 attenuated the growth and metastasis of gastric cancer cells *in vivo*

**Figure 6:**
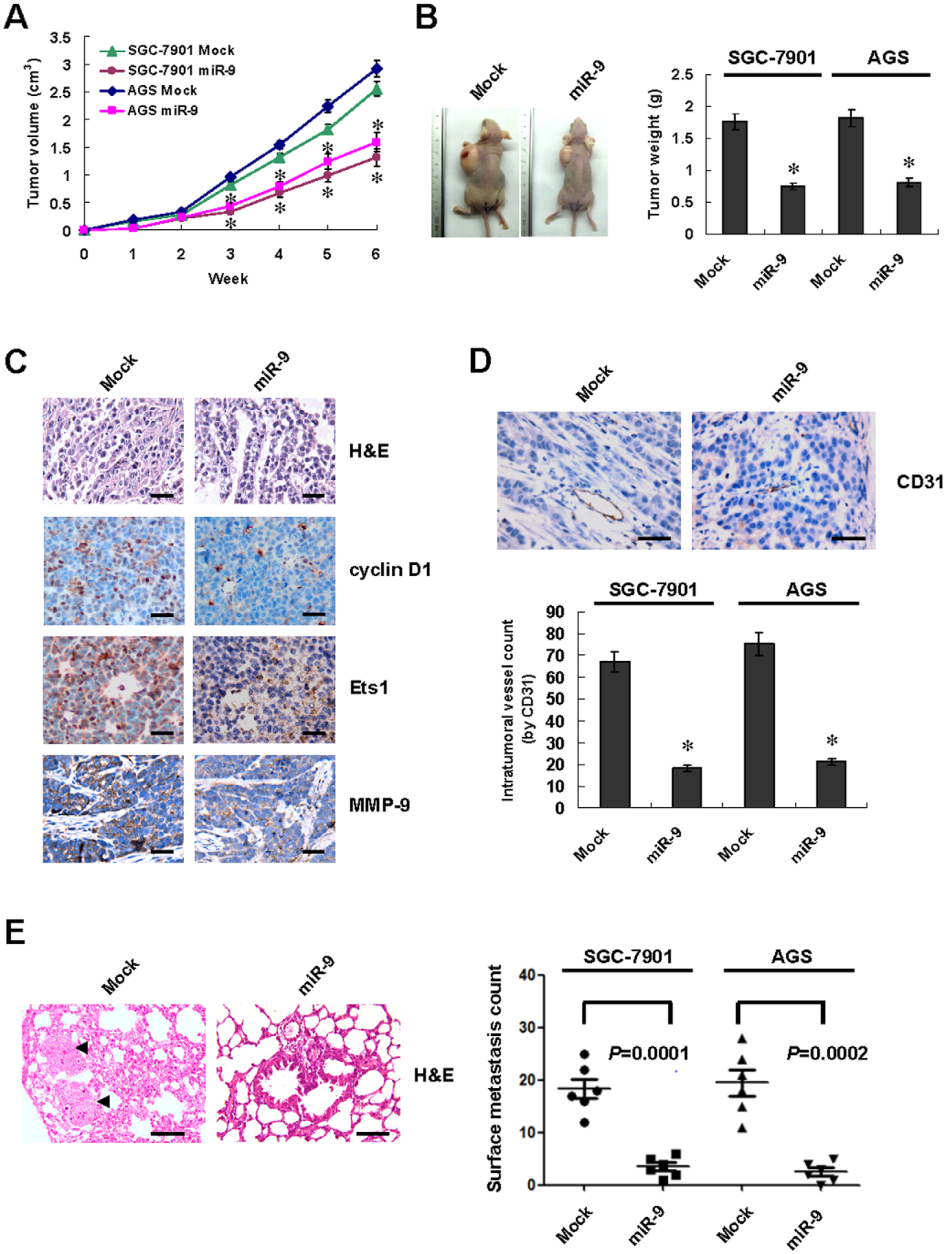
miR-9 attenuated the growth and metastasis of gastric cancer cells *in vivo*. **A** and **B**, hypodermic injection of SGC-7901 or AGS cells stably transfected with miR-9 precursor into athymic nude mice (n = 5) resulted in decreased size and weight of subcutaneous xenograft tumors than those transfected with negative control vector (mock, n = 5). **C**, hematoxylin and eosin (H&E) and immunohistochemical staining revealed that stable transfection of miR-9 precursor resulted in decreased expression of cyclin D1, Ets1, and MMP-9 within tumors. Scale bars: 100 µm. **D**, the CD31-positive mean vessel density was decreased within tumors formed by cancer cells stably transfected by miR-9 precursor. Scale bars: 100 µm. **E**, in the experimental metastasis assay, cancer cells stably transfected with miR-9 precursor established significantly fewer metastatic lung colonies (arrowheads) in athymic nude mice (n = 6). Scale bars: 100 µm. The symbol (*) indicates a significant decrease from mock.

We next investigated the efficacy of miR-9 against tumor growth and metastasis *in vivo*. Stable transfection of miR-9 precursor into SGC-7901 or AGS cells resulted in decreased growth and weight of subcutaneous xenograft tumors in athymic nude mice, when compared to those stably transfected with negative control vector (mock) (Fig. 6A and Fig. 6B). Moreover, the expression of cyclin D1, Ets1 and downstream MMP-9 was reduced by stable transfection of miR-9 precursor (Fig. 6C). The CD31-positive mean vessel density was reduced within tumors formed by injection of cancer cells stably transfected with miR-9 precursor (Fig. 6D). In the experimental metastasis studies, SGC-7901 or AGS cells stably transfected with miR-9 precursor established statistically fewer lung metastatic colonies than mock group (Fig. 6E). These results suggested that miR-9 inhibited the growth and metastasis of gastric cancer cells *in vivo*.

### Knockdown of cyclin D1 and Ets1 phenocopied miR-9 over-expression-mediated inhibition on the proliferation, migration and invasion of gastric cancer cells *in vitro*

**Figure 7:**
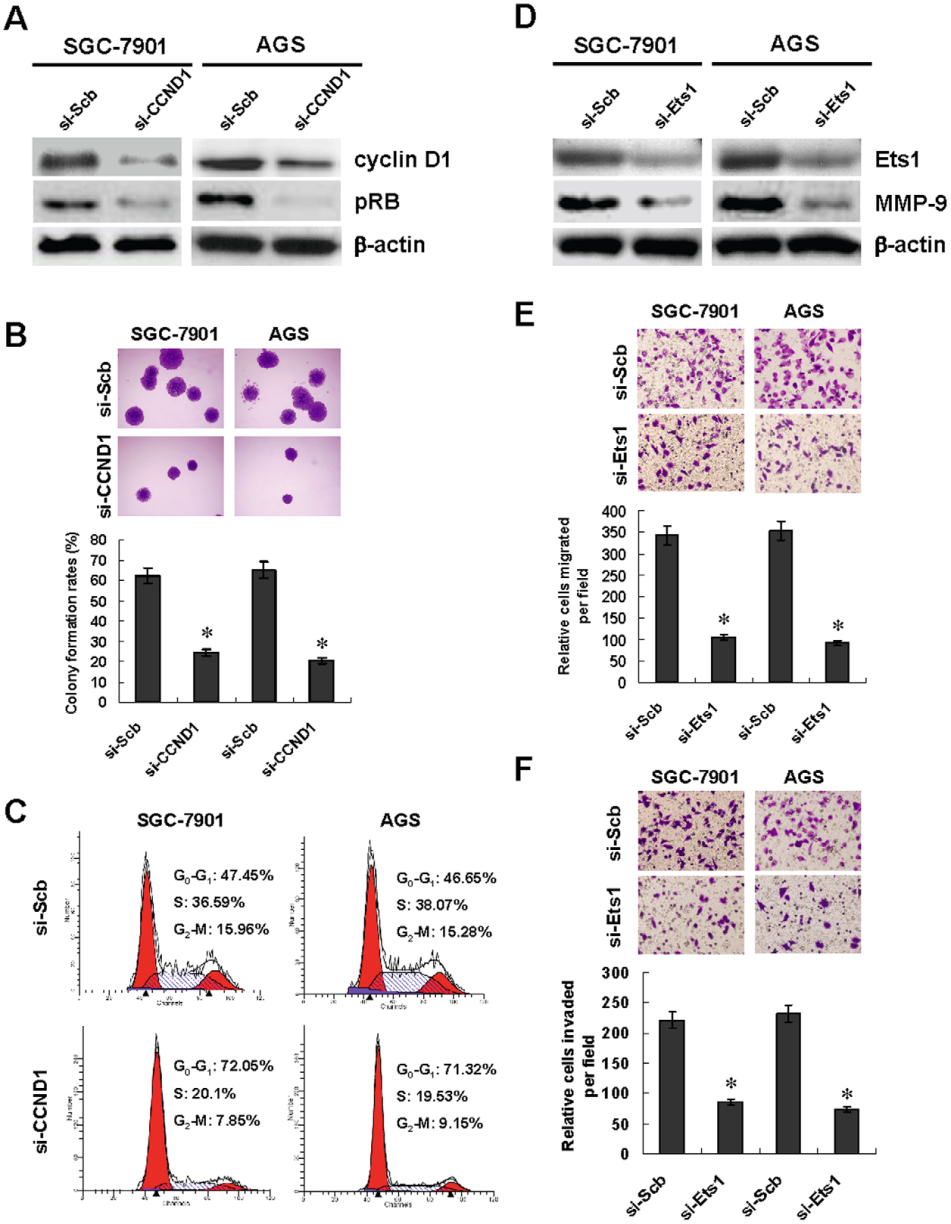
Knockdown of cyclin D1 and Ets1 suppressed the proliferation, migration, and invasion of gastric cancer cells *in vitro*. **A**, transfection of si-CCND1 (100 nmol/L) into gastric cancer SGC-7901 and AGS cells resulted in decreased expression of cyclin D1 and its downstream target pRB than those transfected with scramble siRNA (si-Scb, 100 nmol/L). **B**, knockdown of cyclin D1 suppressed the proliferation of SGC-7901 and AGS cells than those transfected with si-Scb. **C**, knockdown of cyclin D1 induced cell cycle arrest at G_0_/G_1_ phase than those transfected with si-Scb. **D**, transfection of si-Ets1 (100 nmol/L) into gastric cancer cells resulted in decreased expression of Ets1 and its downstream gene MMP-9 than those transfected with si-Scb (100 nmol/L). **E**, the migration capabilities of SGC-7901 and AGS cells were decreased by transfection of si-Ets1 than those transfected with si-Scb. **F**, the invasion capabilities of SGC-7901 and AGS cells were reduced by transfection of si-Ets1 than those transfected with si-Scb. The symbol (*) indicates a significant decrease from si-Scb.

Since above results indicated the negative regulation of cyclin D1 and Ets1 expression by miR-9, we hypothesized that knockdown of cyclin D1 and Ets1 might exert similar effects on cultured gastric cancer cells. The small interfering RNAs (siRNAs) targeting the encoding region of cyclin D1 and Ets1, si-CCND1 and si-Ets1, were designed and transfected into SGC-7901 and AGS cells. Transfection of si-CCND1 and si-Ets1 resulted in decreased expression of cyclin D1, pRB, Ets1 and MMP-9 in gastric cancer cells, respectively, when compared to those transfected with scramble siRNA (si-Scb) (Fig. 7A, Fig. 7D and Fig. S5). Knockdown of cyclin D1 suppressed the proliferation and induced cell cycle arrest at G_0_/G_1_ phase in SGC-7901 and AGS cells (Fig. 7B and Fig. 7C). In addition, knockdown of Ets1 in SGC-7901 and AGS cells resulted in decreased capabilities of migration and invasion (Fig. 7E and Fig. 7F). These results suggested that knockdown of cyclin D1 and Ets1 phenocopied (possessed the similar phenotypes to) miR-9 over-expression in inhibiting the proliferation, migration and invasion of gastric cancer cells *in vitro*.

## Discussion

It has been well established that the miR-9 expression profile in cancer relies on tissue distribution. In primary brain tumors, miR-9 is elevated and functions as an essential factor in neural carcinogenesis [10]. miR-9 is also over-expressed in cervical cancer [11], suggesting its tumor promoter role in tumor development and progression. However, miR-9 is down-regulated in multiple human cancers, including pancreatic cancer [12], ovarian cancer [13] and colorectal cancer [14], and is associated with the malignant progression of breast cancer [25] and colorectal cancer (14). Luo *et al.* noted the down-regulation of miR-9 in 24 gastric cancer specimens [15], which was subsequently confirmed in 9 [16], 72 [17], and 13 [18] gastric cancer cases. However, Inoue *et al.* reported the upregulation of miR-9 in 5 gastric cancer patients by real-time PCR-based miRNA arrays [26], and this different finding in miR-9 expression profile may be due to the heterogeneity of limited specimens. In this study, we demonstrate that miR-9 is significantly down-regulated in 86 primary gastric cancer specimens than in adjacent non-neoplastic tissues, which is consistent with previous findings [15]–[18]. Importantly, we also confirm that miR-9 expression is inversely associated with the clinical stages, invasion, and metastasis of gastric cancer. In humans, the mature miR-9 is encoded by three independent genes, miR-9-1, miR-9-2 and miR-9-3, locating at chromosomes 1, 5 and 15, respectively [27]. Previous studies indicate that the down-regulated miR-9 expression in gastric cancer is due to aberrant hypermethylation of the promoter region of miR-9 encoding genes [17]. Similarly, aberrant hypermethylation of miR-9 family genes is also reported in some primary tumors with lymph node metastasis, such as colon cancer, lung cancer, breast cancer, and melanoma [14], [28]. Importantly, the current study reported the inverse correlation between miR-9 levels and the expression of cyclin D1 and Ets1 in gastric cancer tissues, implying that cyclin D1 and Ets1 may be negatively regulated by miR-9.

The function and target genes of miR-9 are tumor-specific and dependent on cellular context. miR-9 is able to suppress E-cadherin expression in breast cancer [29], resulting in the nuclear translocation of β-catenin and its binding with transcription factors T-cell factor/lymphoid enhancer factor 1, and subsequent up-regulated transcription of genes that facilitate cell proliferation and angiogenesis [29]. Forced miR-9 expression in breast cancer cell lines also results in significant expression changes of multiple genes in the p53-related apoptotic pathway [30]. Through direct targeting NF-κB, ectopic expression of miR-9 inhibits the *in vitro* and *in vivo* growth of ovarian cancer cells [31] and the growth and metastasis of melanoma [32]. In neuroblatoma, over-expression of miR-9 inhibits the invasion, metastasis, and angiogenesis of tumor cells through targeting matrix metalloproteinase 14 [33]. Wan *et al.* reported that over-expression of miR-9 inhibited the *in vitro* and *in vivo* growth of gastric adenocarcinoma cell line MGC803 through repressing NF-κB at the post-transcriptional level [16]. However, in a recent study, Rotkrua *et al.* indicated that miR-9 might be involved in gastric carcinogenesis through down-regulating CDX2, and knockdown of miR-9 decreased the *in vitro* proliferation of gastric cancer MKN-45 cells [19], while their findings need be further strengthened with miR-9 over-expression, target gene rescue, and *in vivo* studies. In addition, it is currently controversial whether CDX2 plays an oncogenic or tumor suppressor role in gastric carcinogenesis [34], [35]. Moreover, the potential roles of miR-9 in the invasion and metastasis of gastric cancer have not been elucidated so far. Thus, the function and direct targets of miR-9 in gastric cancer warrant further investigation.

In this study, we demonstrated that over-expression of miR-9 attenuated the proliferation, migration and invasion of gastric cancer cells, which was similar to that of cyclin D1 or Ets1 knockdown, suggesting the potential application of miR-9 as a target for the therapeutics of gastric cancer. In addition, our data confirmed that miR-9 directly targeted cyclin D1 and Ets1 in gastric cancer cells through 3′-UTR luciferase reporter assay. Since recent evidence shows that certain miRNAs may alternatively modulate gene expression by interacting with promoters [36]–[38], the potential roles of miR-9 in regulating the transcription of cyclin D1 and Ets1 through interacting with promoters remain unclear and warrant our further investigation. Cyclin D1 is a proto-oncogene that belongs to the family of G_1_ cyclins, and plays an important role in cell cycle G_1_ to S transition by binding its partners cyclin dependent kinase 4 and 6 to phosphorylate and inactivate the RB protein [21]. Over-expression of cyclin D1 is an early event in carcinogenesis in human colorectal, esophageal and gallbladder cancers [39], and is a prognostic indicator associated with poor survival in esophageal, breast, and urinary cancers [39]. Cyclin D1 over-expression in human cancers is elucidated by multiple mechanisms, including genomic alterations, post-transcriptional regulation, and post-translational protein stabilization [39]. Recent evidence shows that miR-16-1 represses cyclin D1 expression at the post-transcriptional level via binding its 3′-UTR in mantle cell lymphoma [40]. In the current study, our findings showed that miR-9-mediated inhibition on cell cycle progression and cell proliferation was rescued by restoration of cyclin D1 expression in gastric cancer cells, suggesting that the identification of cyclin D1 as a miR-9 target gene, may explain, at least in part, why over-expression of miR-9 suppressed the proliferation of gastric cancer cells.

Ets1, a member of the ETS family of transcription factors, binds to specific DNA sequences containing a GGAA/T core motif [22], and participates in tumor invasion and metastasis through transcriptional regulation of several genes responsible for extracellular matrix remodeling, migration, and invasion, such as matrix metalloproteinase and urokinase plasminogen activator [22]. To date, an increasing number of clinical studies have shown the important roles of Ets1 in tumor development and progression of various solid tumors [23], [24]. Previous studies indicate that Ets1 expression is related to the clinicopathological features of gastric cancer, including tumor infiltration and lymph nodes or distal metastasis, suggesting its critical role in the invasion and metastasis of gastric cancer [41]. However, the mechanisms underlying the Ets1 over-expression in gastric cancer still remains largely unknown. Recent studies reveal the post-transcriptional regulation of Ets1 by miR-125b in breast cancer cells [42], and by miR-200b in endothelial cells [43]. In this study, we demonstrated that as a direct target of miR-9, Ets1 was up-regulated in gastric cancer and contributed to the migration and invasion of cancer cells. Knockdown of Ets1 partially phenocopied the effects of miR-9 over-expression in gastric cancer cell lines, while restoration of Ets1 expression rescued the cancer cells from miR-9-mediated inhibition on the migration and invasion, revealing a novel post-transcriptional regulation mechanism of Ets1 by miR-9 and its clinical potentials in gastric cancer.

In summary, we have demonstrated, for the first time, that miR-9 represses the expression of cyclin D1 and Ets1 through directly targeting their 3′-UTR, thus inhibiting the proliferation, invasion and metastasis of gastric cancer cells. This study extends our knowledge about the regulation of cyclin D1 and Ets1 at the post-transcriptional level by miRNA, and suggests that miR-9 may be of potential values as a novel therapeutic target for gastric cancer.

## Materials and Methods

### Patient tissue samples

Approval to conduct this study was obtained from the Institutional Review Board of Tongji Medical College (approval number: 2010-S003). Paraffin-embedded and fresh specimens of 86 primary gastric cancer cases were obtained from the Department of Surgery, Union Hospital of Tongji Medical College. Their pathological diagnosis was proved by at least two pathologists. The demographic and clinicopathological data of all patients were summarized in Table S1. Adjacent gastric mucosa that contained no macroscopic tumor was also obtained, and the non-neoplastic areas were subsequently verified by microscopic histology. The fresh tumor specimens and adjacent non-neoplastic mucosa were collected and stored at −80°C until use.

### Immunohistochemistry

Immunohistochemical staining was performed as previously described [44], with antibodies specific for cyclin D1, Ets1, MMP-9 and CD31 (Abcam Inc, Cambridge, MA, 1:200 dilutions). The negative controls included parallel sections treated with omission of the primary antibody, in addition to an adjacent section of the same block in which the primary antibody was replaced by rabbit polyclonal IgG (Abcam Inc) as an isotype control. The reactivity degree was assessed by at least two pathologists without knowledge of the clinicopathological features of tumors. The degree of positivity was initially classified according to the percentage of positive cancer cells as the following: (−) <5% cells positive, (1+) 6–25% cells positive, (2+) 26–50% cells positive, and (3+) >50% cells positive. Slides with moderate positive (2+) or strong positive (3+) reactivity were classified as having a “high expression”, whereas slides with negative (−) or weak positive (1+) reactivity were classified as having a “low expression”.

### Western blot

Cellular protein was extracted with 1× cell lysis buffer (Promega, Madison, WI). Protein (50 µg) from each sample was subjected to 4–20% pre-cast polyacrylamide gel (Bio-Rad, Hercules, CA) electrophoresis and transferred to nitrocellulose membranes (Bio-Rad). For cyclin D1, Ets1, MMP-9, pRB and β-actin (Santa Cruz Biotechnology, Santa Cruz, CA) detection, the primary antibody dilutions were 1∶500, 1∶500, 1∶500, 1∶500 and 1∶1000, respectively, followed by 1∶3000 dilution of goat anti-rabbit horseradish peroxidase-labeled antibody (Bio-Rad). Enhanced chemiluminescence substrate kit (Amersham, Piscataway, NJ) was used for the chemiluminscent detection of signals with autoradiography film (Amersham).

### RT-PCR and real-time quantitative RT-PCR

Total RNA was isolated with RNeasy Mini Kit (Qiagen Inc., Valencia, CA). The reverse transcription reactions were conducted with Transcriptor First Strand cDNA Synthesis Kit (Roche, Indianapolis, IN). The PCR primers for cyclin D1, Ets1, MMP-9 and β-actin were designed by Premier Primer 5.0 software (Table S2). RT-PCR was performed as previously described [44]. Real-time quantitative RT-PCR with SYBR Green PCR Master Mix (Applied Biosystems, Foster City, CA) was performed using ABI Prism 7700 Sequence Detector (Applied Biosystems). The fluorescent signals were collected during extension phase, Ct values of the samples were calculated, and the transcript levels of cyclin D1, Ets1 and MMP-9 were normalized to those of β-actin by 2^−ΔΔCt^ method.

### Quantification of miR-9 expression

The levels of mature miR-9 in primary tissues and cell lines were determined using Bulge-Loop™ miRNAs qPCR Primer Set (RiboBio Co. Ltd, Guangzhou, China). After cDNA was synthesized with a miRNA-specific stem-loop primer, the quantitative PCR was performed with the specific primers (Table S2). The miR-9 levels were normalized to those of U6 snRNA.

### miRNA target prediction

miRNA targets were predicted using the algorithms miRanda, miRDB, RNA22, and Targetscan [45]. To identify the genes commonly predicted by four different algorithms, the results of predicted targets were intersected using miRWalk [46].

### Cell culture and transfection

Human gastric cancer cell lines (SGC-7901 and MKN-74) and SV40-transformed, normal and non-tumorigenic gastric epithelial GES-1 cells [20] were obtained from the Type Culture Collection of Chinese Academy of Sciences (Shanghai, China). Human gastric cancer cell line AGS (CRL-1739) was purchased form American Type Culture Collection (Rockville, MD). Cells were grown in RPMI1640 medium (Life Technologies, Inc., Gaithersburg, MD) supplemented with 10% fetal bovine serum (Life Technologies, Inc.), penicillin (100 U/ml) and streptomycin (100 µg/ml). Cells were maintained at 37°C in a humidified atmosphere of 5% CO_2_. Anti-miR-9 or negative control inhibitors (RiboBio Co. Ltd) were transfected into confluent cells with Lipofectamine 2000 (Life Technologies, Inc.), according to the manufacturer's instructions.

### pre-miR-9 construct and stable transfection

According to the pre-miR-9 (5′-TCTTTGGTTATCTAGCTGTATGA-3′) sequence documented in miRNA Registry database, oligonucleotides encoding miR-9 precursor (Table S3) were subcloned into linearized pcDNA6.2-GW/EmGFP-miR with 4 nucleotide overhangs (Invitrogen, Carlsbad, CA), and verified by DNA sequencing (GenePharma Co., Ltd, Shanghai, China). A negative control vector was established with an insert (Table S3) that forms a hairpin structure and processes into mature miRNA, but does not target any known vertebrate gene (Invitrogen). The negative control and pcDNA6.2-GW/EmGFP-miR9 vectors were transfected into cancer cells, and stable cell lines were screened by administration of Blasticidin (Invitrogen).

### Luciferase reporter assay

Human cyclin D1 3′-UTR (3192 bp) and Ets1 3′-UTR (3519 bp) containing the putative binding sites of miR-9 were amplified by PCR (Table S3), inserted into the firefly luciferase reporter vector pmiR-RB-REPORT™ (RiboBio Co. Ltd) between the restrictive sites *Xho* I and *Not* I, and validated by sequencing. Their mutant constructs with a mutation of the miR-9 seed sequence were generated with the mutagenic oligonucleotide primers (Table S3), according to the manual of GeneTailor Site-Directed Mutagenesis System (Invitrogen). Cells were plated at 1×10^5^ cells/well on 24-well plates, and transfected with pmiR-RB-cyclin D1 3′-UTR (30 ng), pmiR-RB-Ets1 3′-UTR (30 ng) or their mutant constructs. Twenty-four hrs post-transfection, firefly and *Renilla* luciferase activities were consecutively measured, according to the dual-luciferase assay manual (Promega). The *Renilla* luciferase signal was normalized to the firefly luciferase signal for each individual analysis.

### Restoration and knockdown of cyclin D1 or Ets1

Human cyclin D1 cDNA (888 bp) and Ets1 cDNA (1458 bp) were amplified from gastric cancer tissue (Table S3), subcloned into pcDNA3.1/Zeo(+) (Invitrogen), and validated by sequencing. To rescue the miR-9-induced down-regulation of target genes, stable cell lines were transfected with the recombinant vector pcDNA3.1-cyclin D1 or pcDNA3.1-Ets1 using Lipofectamine 2000. The 21-nucleotide siRNAs targeting the encoding region of cyclin D1 and Ets1 were chemically synthesized (RiboBio Co. Ltd) and transfected with Genesilencer Transfection Reagent (Genlantis, San Diego, CA). The si-Scb was applied as controls (Table S3).

### Colony formation assay

Cell lines were seeded at a density of 300/ml on 35-mm dishes. Colony formation assay was performed as previously described [47]. Positive colony formation (more than 50 cells/colony) was counted. The survival fraction of cells was expressed as the ratio of plating efficiency of treated cells to that of untreated control cells.

### Cell cycle assay

Cell cycles were examined by flow cytometry [48]. Briefly, cell lines underwent starvation for 12 hrs for synchronization, and 2×10^5^ cells were collected and fixed in 70% ethanol overnight at 4°C. Then, cells were washed once with phosphate buffer solution, digested by 200 µl of RNase (1 mg/ml) at 37°C for 30 min, and stained with 800 µl of propidium iodide (50 µg/ml, Sigma, St. Louis, MO) at room temperature for 30 min. The DNA histograms were generated with a flow cytometer (Becton Dickson Co., San Jose, CA).

### Cell migration and invasion assay

The migration assay was performed with Transwell inserts that have 6.5-mm polycarbonate membranes with 8.0-µm pores (Corning, New York, NY). Matrigel invasion assay was undertaken using membranes coated with Matrigel matrix (BD Science, Sparks, MD). Homogeneous single cell suspensions (1 × 10^5^ cells/well) were added to the upper chambers and allowed to invade for 24 hrs at 37°C in a CO_2_ incubator. Migrated or invaded cells were stained with 0.1% crystal violet for 10 min at room temperature and examined by light microscopy. Quantification of migrated or invaded cells was performed according to published criteria [49].

### *In vivo* growth and metastasis assay

All animal experiments were approved by the Animal Care Committee of Tongji Medical College (approval number: Y20080290). For the *in vivo* tumor growth studies, 2-month-old male nude mice (n = 5 per group) were injected subcutaneously in the upper back with 1×10^6^ cancer cells stably transfected with negative control or miR-9 precursor vectors. Six weeks later, mice were sacrificed and examined for tumor weight, gene expression, and angiogenesis. The experimental metastasis (0.4×10^6^ cancer cells per mouse) studies were performed with 5-week-old male nude mice as previously described [50].

### Statistical analysis

Unless otherwise stated, all data were shown as mean ± standard error of the mean (SEM). The SPSS 18.0 statistical software (SPSS Inc., Chicago, IL) was applied for statistical analysis. The χ^2^ analysis and Fisher exact probability analysis were applied for comparison among the expression of cyclin D1, Ets1, miR-9 and individual clinicopathological feature. Pearson's coefficient correlation was applied for analyzing the relationship between miR-9 and transcripts of cyclin D1 or Ets1. Difference of tissue specimens or cancer cells was determined by *t* test or analysis of variance (ANOVA), and considered significant when the *P* value was less than 0.05.

## Supporting Information

Figure S1 **Immunohistochemical staining of cyclin D1 and Ets1 in gastric cancer specimens.** Nuclear cyclin D1 (arrowheads) and nuclear (arrowheads) or cytoplasm staining of Ets1 were noted in cancer cells of gastric cancer tissues. However, low cyclin D1 immunostaining and no obvious immunostaining of Ets1 were observed in adjacent non-neoplastic mucous. Scale bars: 100 µm.
(PDF)

Figure S2 **Ectopic expression of miR-9 in gastric cancer cells.** Real-time quantitative RT-PCR indicated that stable transfection of miR-9 precursor into gastric cancer SGC-7901 and AGS cells, resulted in enhanced miR-9 levels than those transfected with negative control vector (mock). The symbol (#) indicates a significant increase from mock.
(PDF)

Figure S3 **Knockdown of miR-9 promoted the proliferation, migration and invasion of cultured cell lines.** Transfection of anti-miR-9 inhibitor (100 nmol/L) into normal gastric epithelial GES-1 cells and gastric cancer SGC-7901 and AGS cells resulted in decreased miR-9 expression (**A**), increased proliferation (**B**), promoted G_1_/S phase transition (**C**), enhanced migration (**D**), and increased invasiveness (**E**), when compared to those transfected with negative control inhibitor (anti-NC, 100 nmol/L). The symbols (* and #) indicate a significant decrease and a significant increase from anti-NC, respectively.
(PDF)

Figure S4 **Knockdown of miR-9 directly enhanced the expression of cyclin D1 and Ets1 in GES-1 cells.** When compared to those transfected with negative control inhibitor (anti-NC, 100 nmol/L), transfection of anti-miR-9 inhibitor (100 nmol/L) into normal gastric epithelial GES-1 cells decreased the protein (**A**) and mRNA (**B**) levels of cyclin D1, Ets1 and their downstream genes (pRB and MMP-9), and these effects were exerted through direct binding with intact miR-9 binding site (Wt) but not its mutation sequence (Mut) (**C**). The symbol (#) indicates a significant increase from anti-NC.
(PDF)

Figure S5 **Knockdown of cyclin D1 and Ets1 in gastric cancer cells.** Real-time quantiative RT-PCR indicated that transfection of si-CCND1 (100 nmol/L) and si-Ets1 (100 nmol/L) into gastric cancer SGC-7901 and AGS cells resulted in decreased transcript levels of cyclin D1 (**A**), Ets1 and its downstream gene MMP-9 (**B**) than those transfected with scramble siRNA (si-Scb, 100 nmol/L). The symbol (*) indicates a significant decrease from si-Scb.
(PDF)

Table S1 **Cyclin D1, Ets1 and miR-9 expression in human gastric cancer tissues.**
(PDF)

Table S2 **Primer sets used for RT-PCR and qPCR.**
(PDF)

Table S3 **Oligonucleotide sets used for constructs, miRNA inhibitor and small interfering RNAs.**
(PDF)

## References

[1] Terry MB, Gaudet MM, Gammon MD (2002) The epidemiology of gastric cancer. Semin Radiat Oncol 12: 111–127. 11979413 10.1053/srao.30814

[2] Noguchi T, Wirtz HC, Michaelis S, Gabbert HE, Mueller W (2001) Chromosomal imbalances in gastric cancer. Correlation with histologic subtypes and tumor progression. Am J Clin Pathol 115: 828–834. 11392878 10.1309/2Q9E-3EP5-KYPK-VFGQ

[3] Iorio MV, Croce CM (2012) MicroRNA dysregulation in cancer: diagnostics, monitoring and therapeutics. A comprehensive review. EMBO Mol Med 4: 143–159. 22351564 10.1002/emmm.201100209 PMC3376845

[4] Zhang Z, Li Z, Gao C, Chen P, Chen J, et al (2008) miR-21 plays a pivotal role in gastric cancer pathogenesis and progression. Lab Invest 88: 1358–1366. 18794849 10.1038/labinvest.2008.94

[5] Xia L, Zhang D, Du R, Pan Y, Zhao L, et al (2008) miR-15b and miR-16 modulate multidrug resistance by targeting BCL2 in human gastric cancer cells. Int J Cancer 123: 372–379. 18449891 10.1002/ijc.23501

[6] Wang HJ, Ruan HJ, He XJ, Ma YY, Jiang XT, et al (2010) MicroRNA-101 is down-regulated in gastric cancer and involved in cell migration and invasion. Eur J Cancer 46: 2295–2303. 20712078 10.1016/j.ejca.2010.05.012

[7] Li Y, Wang F, Lee JA, Gao FB (2006) MicroRNA-9a ensures the precise specification of sensory organ precursors in Drosophila. Genes Dev 20: 2793–2805. 17015424 10.1101/gad.1466306 PMC1619947

[8] Leucht C, Stigloher C, Wizenmann A, Klafke R, Folchert A, et al (2008) MicroRNA-9 directs late organizer activity of the midbrain-hindbrain boundary. Nat Neurosci 11: 641–648. 18454145 10.1038/nn.2115

[9] Shibata M, Kurokawa D, Nakao H, Ohmura T, Aizawa S (2008) MicroRNA-9 modulates cajal–retzius cell differentiation by suppressing Foxg1 expression in mouse medial pallium. J Neurosci 28: 10415–10421. 18842901 10.1523/JNEUROSCI.3219-08.2008 PMC6671033

[10] Nass D, Rosenwald S, Meiri E, Gilad S, Tabibian-Keissar H, et al (2009) MiR-92b and miR-9/9* are specifically expressed in brain primary tumors and can be used to differentiate primary from metastatic brain tumors. Brain Pathol 19: 375–383. 18624795 10.1111/j.1750-3639.2008.00184.x PMC2728890

[11] Wilting SM, Snijders PJF, Verlaat W, Jaspers A, van de Wiel MA, et al (2012) Altered microRNA expression associated with chromosomal changes contributes to cervical carcinogenesis. Oncogene doi: 10.1038/onc.2012.20. 10.1038/onc.2012.20 22330141

[12] Omura N, Li CP, Li A, Hong SM, Walter K, et al (2008) Genome-wide profiling at methylated promoters in pancreatic adenocarcinoma. Cancer Biol Ther 7: 1146–1156. 18535405 10.4161/cbt.7.7.6208 PMC2763640

[13] Laios A, O'Toole S, Flavin R, Martin C, Kelly L, et al (2008) Potential role of miR-9 and miR-223 in recurrent ovarian cancer. Mol Cancer 7: 35. 18442408 10.1186/1476-4598-7-35 PMC2383925

[14] Bandres E, Agirre X, Bitarte N, Ramirez N, Zarate R, et al (2009) Epigenetic regulation of microRNA expression in colorectal cancer. Int J Cancer 125: 2737–2743. 19521961 10.1002/ijc.24638

[15] Luo H, Zhang H, Zhang Z, Zhang X, Ning B, et al (2009) Down-regulated miR-9 and miR-433 in human gastric carcinoma. J Exp Clin Cancer Res 28: 82. 19531230 10.1186/1756-9966-28-82 PMC2739520

[16] Wan HY, Guo LM, Liu T, Liu M, Li X, et al (2010) Regulation of the transcription factor NF-kappaB1 by microRNA-9 in human gastric adenocarcinoma. Mol Cancer 9: 16. 20102618 10.1186/1476-4598-9-16 PMC2835654

[17] Tsai KW, Liao YL, Wu CW, Hu LY, Li SC, et al (2011) Aberrant hypermethylation of miR-9 genes in gastric cancer. Epigenetics 6: 1189–1197. 21931274 10.4161/epi.6.10.16535 PMC3225840

[18] Saito Y, Suzuki H, Taya T, Nishizawa M, Tsugawa H, et al (2012) Development of a novel microRNA promoter microarray for ChIP-on-chip assay to identify epigenetically regulated microRNAs. Biochem Biophys Res Commun 426: 33–37. 22906743 10.1016/j.bbrc.2012.08.012

[19] Rotkrua P, Akiyama Y, Hashimoto Y, Otsubo T, Yuasa Y (2011) MiR-9 downregulates CDX2 expression in gastric cancer cells. Int J Cancer 129: 2611–2620. 21225631 10.1002/ijc.25923

[20] Ke Y, Ning T, Wang B (1994) Establishment and characterization of a SV40 transformed human fetal gastric epithelial cell line-GES-1. Zhonghua Zhong Liu Za Zhi 16: 7–10. 8033753

[21] Kato J, Matsushime H, Hiebert SW, Ewen ME, Sherr CJ (1993) Direct binding of cyclin D to the retinoblastoma gene product (pRb) and pRb phosphorylation by the cyclin D-dependent kinase CDK4. Genes Dev 7: 331–342. 8449399 10.1101/gad.7.3.331

[22] Sahin A, Velten M, Pietsch T, Knuefermann P, Okuducu AF, et al (2005) Inactivation of Ets 1 transcription factor by a specific decoy strategy reduces rat C6 glioma cell proliferation and mmp-9 expression. Int J Mol Med 15: 771–776. 15806297

[23] Endo K, Shirai A, Furukawa M, Yoshizaki T (2006) Prognostic value of cell motility activation factors in patients with tongue squamous cell carcinoma. Hum Pathol 37: 1111–1116. 16867875 10.1016/j.humpath.2006.03.020

[24] Nakayama T, Ito M, Ohtsuru A, Naito S, Sekine I (2001) Expression of the ets-1 proto-oncogene in human colorectal carcinoma. Mod Pathol 14: 415–422. 11353051 10.1038/modpathol.3880328

[25] Iorio MV, Ferracin M, Liu C-G, Veronese A, Spizzo R, et al (2005) MicroRNA gene expression deregulation in human breast cancer. Cancer Res 65: 7065–7070. 16103053 10.1158/0008-5472.CAN-05-1783

[26] Inoue T, Iinuma H, Ogawa E, Inaba T, Fukushima R (2012) Clinicopathological and prognostic significance of microRNA-107 and its relationship to DICER1 mRNA expression in gastric cancer. Oncol Rep 27: 1759–1764. 22407237 10.3892/or.2012.1709

[27] Hildebrandt MAT, Gu J, Lin J, Ye Y, Tan W, et al (2010) Hsa-miR-9 methylation status is associated with cancer development and metastatic recurrence in patients with clear cell renal cell carcinoma. Oncogene 29: 5724–5728. 20676129 10.1038/onc.2010.305

[28] Lujambio A, Calin GA, Villanueva A, Ropero S, Sánchez-Céspedes M, et al (2008) A microRNA DNA methylation signature for human cancer metastasis. Proc Natl Acad Sci USA 105: 13556–13561. 18768788 10.1073/pnas.0803055105 PMC2528872

[29] Ma L, Young J, Prabhala H, Pan E, Mestdagh P, et al (2010) miR-9, a MYC/MYCN -activated microRNA, regulates E-cadherin and cancer metastasis. Nat Cell Biol 12: 247–256. 20173740 10.1038/ncb2024 PMC2845545

[30] Hsu PY, Deatherage DE, Rodriguez BAT, Liyanarachchi S, Weng YI, et al (2009) Xenoestrogen-induced epigenetic repression of microRNA-9-3 in breast epithelial cells. Cancer Res 69: 5936–5945. 19549897 10.1158/0008-5472.CAN-08-4914 PMC2855843

[31] Guo LM, Pu Y, Han Z, Liu T, Li YX, et al (2009) MicroRNA-9 inhibits ovarian cancer cell growth through regulation of NF-kappaB1. FEBS J 276: 5537–5546. 19702828 10.1111/j.1742-4658.2009.07237.x

[32] Liu S, Kumar SM, Lu H, Liu A, Yang R, et al (2012) MicroRNA-9 up-regulates E-cadherin through inhibition of NF-κB1-Snail1 pathway in melanoma. J Pathol 226: 61–72. 22131135 10.1002/path.2964 PMC3959162

[33] Zhang H, Qi M, Li S, Qi T, Mei H, et al (2012) microRNA-9 Targets Matrix Metalloproteinase 14 to Inhibit Invasion, Metastasis, and Angiogenesis of Neuroblastoma Cells. Mol Cancer Ther 11: 1454–1466. 22564723 10.1158/1535-7163.MCT-12-0001

[34] Jang BG, Kim WH (2011) Molecular pathology of gastric carcinoma. Pathobiology 78: 302–310. 22104201 10.1159/000321703

[35] Wang XT, Xie YB, Xiao Q (2012) siRNA targeting of Cdx2 inhibits growth of human gastric cancer MGC-803 cells. World J Gastroenterol 18: 1903–1914. 22563170 10.3748/wjg.v18.i16.1903 PMC3337565

[36] Place RF, Li LC, Pookot D, Noonan EJ, Dahiya R (2008) MicroRNA-373 induces expression of genes with complementary promoter sequences. Proc Natl Acad Sci USA 105: 1608–1613. 18227514 10.1073/pnas.0707594105 PMC2234192

[37] Kim DH, Saetrom P, Snøve O Jr, Rossi JJ (2008) MicroRNA-directed transcriptional gene silencing in mammalian cells. Proc Natl Acad Sci USA 105: 16230–16235. 18852463 10.1073/pnas.0808830105 PMC2571020

[38] Younger ST, Corey DR (2011) Transcriptional gene silencing in mammalian cells by miRNA mimics that target gene promoters. Nucleic Acids Res 39: 5682–5691. 21427083 10.1093/nar/gkr155 PMC3141263

[39] Kim JK, Diehl JA (2009) Nuclear cyclin D1: An oncogenic driver in human cancer. J Cell Physiol 220: 292–296. 19415697 10.1002/jcp.21791 PMC2874239

[40] Chen RW, Bemis LT, Amato CM, Myint H, Tran H, et al (2008) Truncation in CCND1 mRNA alters miR-16-1 regulation in mantle cell lymphoma. Blood 112: 822–829. 18483394 10.1182/blood-2008-03-142182 PMC2481543

[41] Yu Y, Zhang YC, Zhang WZ, Shen LS, Hertzog P, et al (2003) Ets1 as a marker of malignant potential in gastric carcinoma. World J Gastroenterol 9: 2154–2159. 14562368 10.3748/wjg.v9.i10.2154 PMC4656453

[42] Zhang Y, Yan LX, Wu QN, Du ZM, Chen J, et al (2011) miR-125b is methylated and functions as a tumor suppressor by regulating the ETS1 proto-oncogene in human invasive breast cancer. Cancer Res 71: 3552–3562. 21444677 10.1158/0008-5472.CAN-10-2435

[43] Chan YC, Khanna S, Roy S, Sen CK (2011) miR-200b targets Ets-1 and is down-regulated by hypoxia to induce angiogenic response of endothelial cells. J Biol Chem 286: 2047–2056. 21081489 10.1074/jbc.M110.158790 PMC3023502

[44] Jiang G, Zheng L, Pu J, Mei H, Zhao J, et al (2012) Small RNAs targeting transcription start site induce heparanase silencing through interference with transcription initiation in human cancer cells. PLoS ONE 2012 7: e31379. 10.1371/journal.pone.0031379 PMC3282686 22363633

[45] Dai Y, Zhou X (2010) Computational methods for the identification of microRNA targets. Open Access Bioinformatics 2: 29–39. 22162940 10.2147/OAB.S6902 PMC3233190

[46] Dweep H, Sticht C, Pandey P, Gretz N (2011) miRWalk – Database: Prediction of possible miRNA binding sites by “walking” the genes of three genomes. J Biomed Inform 44: 839–847. 21605702 10.1016/j.jbi.2011.05.002

[47] Zheng L, Jiang G, Mei H, Pu J, Dong J, et al (2010) Small RNA interference-mediated gene silencing of heparanase abolishes the invasion, metastasis and angiogenesis of gastric cancer cells. BMC Cancer 10: 33. 20137078 10.1186/1471-2407-10-33 PMC2834619

[48] Tong QS, Jiang GS, Zheng LD, Tang ST, Cai JB, et al (2008) Methyl jasmonate downregulates expression of proliferating cell nuclear antigen and induces apoptosis in human neuroblastoma cell lines. Anti-Cancer Drugs 19: 573–581. 18525316 10.1097/CAD.0b013e3282fc46b0

[49] Valster A, Tran NL, Nakada M, Berens ME, Chan AY, et al (2005) Cell migration and invasion assays. Methods 37: 208–215. 16288884 10.1016/j.ymeth.2005.08.001

[50] Zheng B, Liang L, Wang C, Huang S, Cao X, et al (2011) MicroRNA-148a suppresses tumor cell invasion and metastasis by downregulating ROCK1 in gastric cancer. Clin Cancer Res 17: 7574–7583. 21994419 10.1158/1078-0432.CCR-11-1714

