# Supplementary figures and images for: microRNA-9 Suppresses the Proliferation, Invasion and Metastasis of Gastric Cancer Cells through Targeting Cyclin D1 and Ets1
Source: PLoS One. 2013 Jan 31;8(1):e55719. doi: 10.1371/journal.pone.0055719 (PMC3561302; doi:10.1371/journal.pone.0055719)

# Supplementary Figure S1

Adjacent non-neoplastic

Intestine-type

Diffuse-type

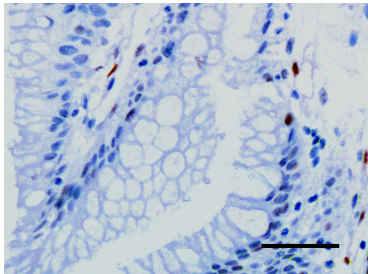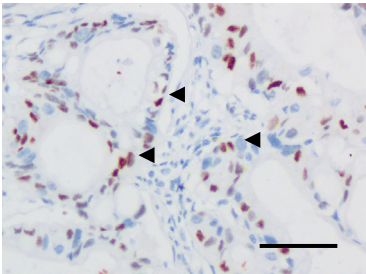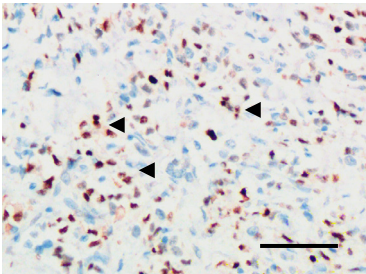

cyclin D1

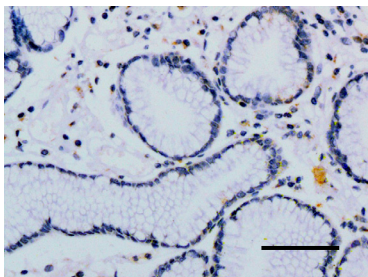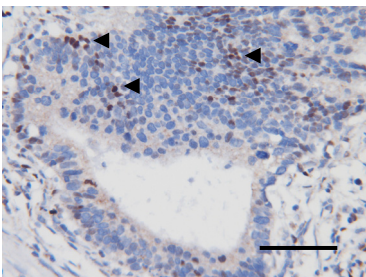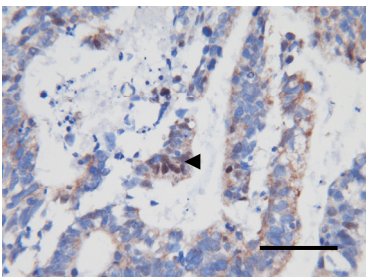

Ets1

Supplement: Figure S1 — Immunohistochemical staining of cyclin D1 and Ets1 in gastric cancer specimens. Nuclear cyclin D1 (arrowheads) and nuclear (arrowheads) or cytoplasm staining of Ets1 were noted in cancer cells of gastric cancer tissues. However, low cyclin D1 immunostaining and no obvious immunostaining of Ets1 were observed in adjacent non-neoplastic mucous. Scale bars: 100 µm. (PDF) [file pone.0055719.s001.pdf]

# Supplementary Figure S2

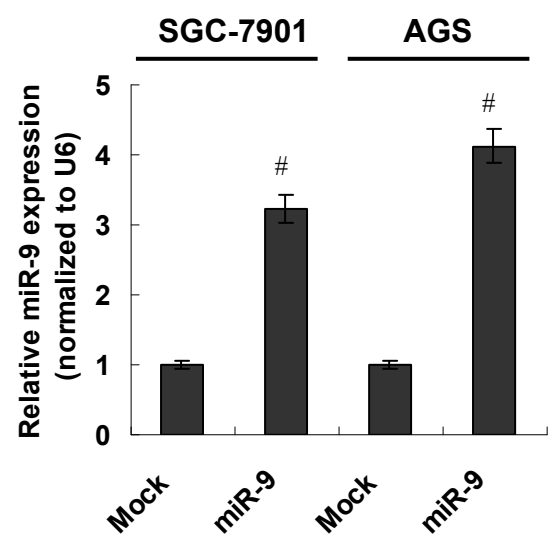

Supplement: Figure S2 — Ectopic expression of miR-9 in gastric cancer cells. Real-time quantitative RT-PCR indicated that stable transfection of miR-9 precursor into gastric cancer SGC-7901 and AGS cells, resulted in enhanced miR-9 levels than those transfected with negative control vector (mock). The symbol (#) indicates a significant increase from mock. (PDF) [file pone.0055719.s002.pdf]

# Supplementary Figure S3

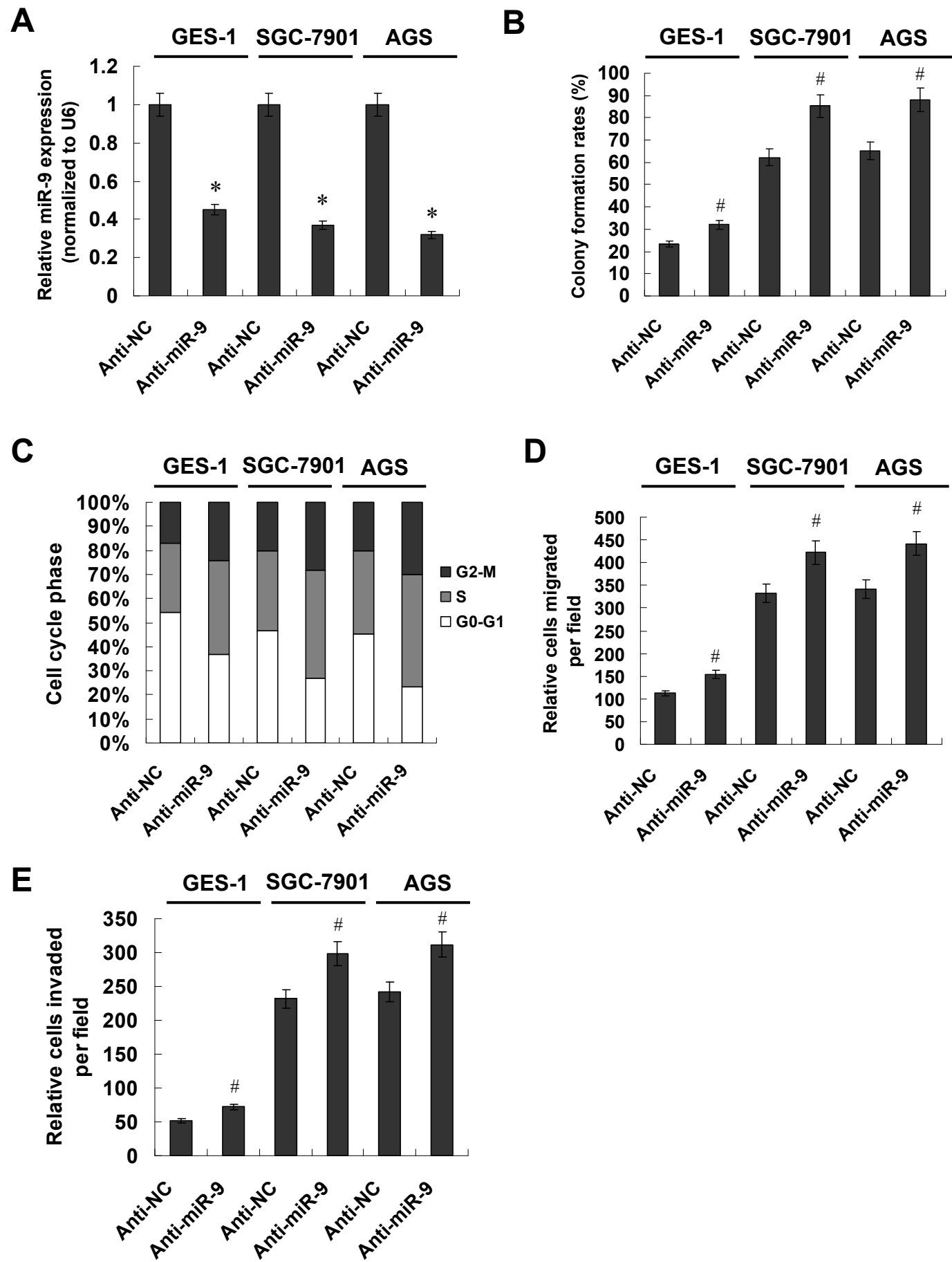

Supplement: Figure S3 — Knockdown of miR-9 promoted the proliferation, migration and invasion of cultured cell lines. Transfection of anti-miR-9 inhibitor (100 nmol/L) into normal gastric epithelial GES-1 cells and gastric cancer SGC-7901 and AGS cells resulted in decreased miR-9 expression (A), increased proliferation (B), promoted G1/S phase transition (C), enhanced migration (D), and increased invasiveness (E), when compared to those transfected with negative control inhibitor (anti-NC, 100 nmol/L). The symbols (* and #) indicate a significant decrease and a significant increase from anti-NC, respectively. (PDF) [file pone.0055719.s003.pdf]

# Supplementary Figure S4

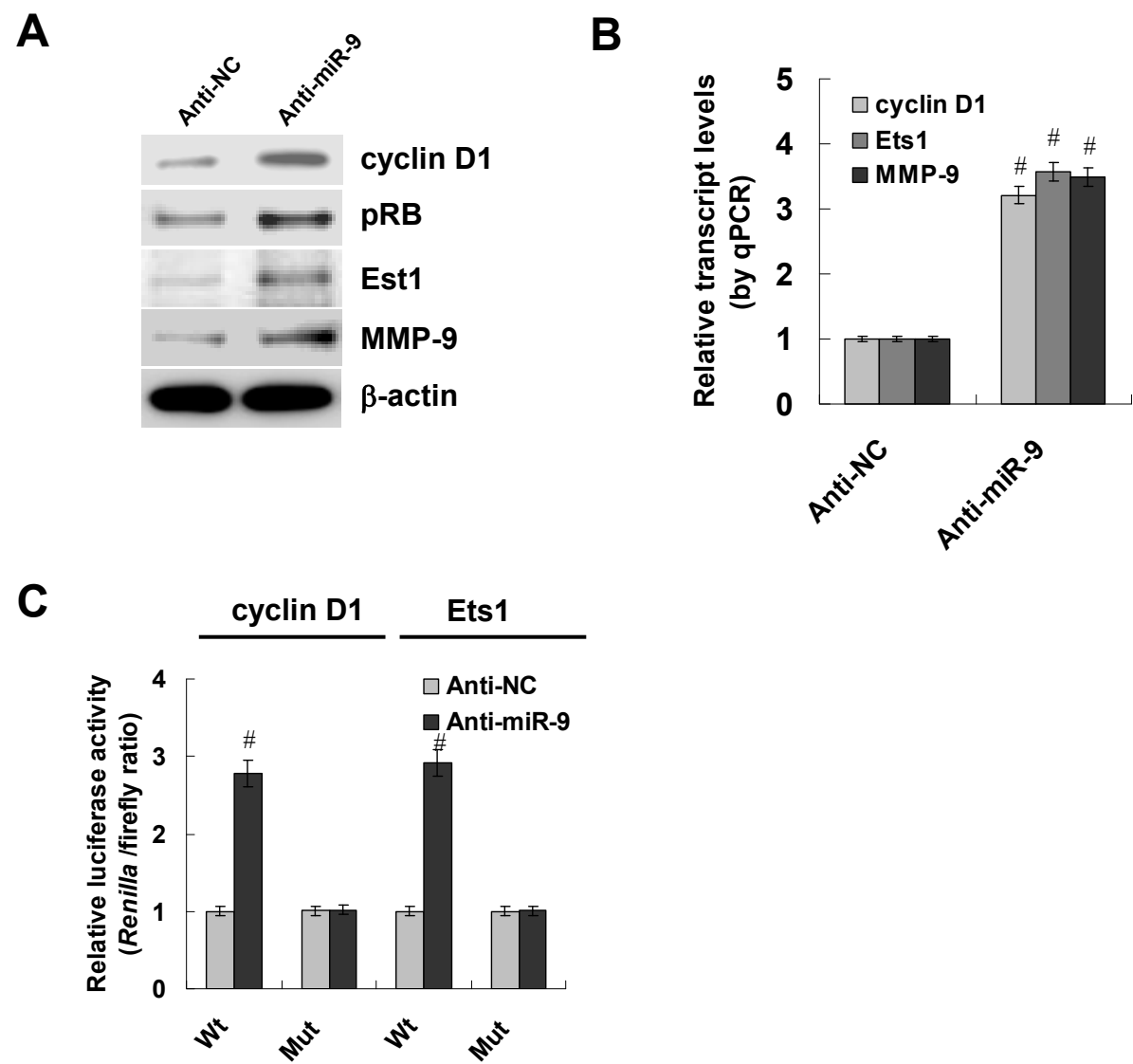

Supplement: Figure S4 — Knockdown of miR-9 directly enhanced the expression of cyclin D1 and Ets1 in GES-1 cells. When compared to those transfected with negative control inhibitor (anti-NC, 100 nmol/L), transfection of anti-miR-9 inhibitor (100 nmol/L) into normal gastric epithelial GES-1 cells decreased the protein (A) and mRNA (B) levels of cyclin D1, Ets1 and their downstream genes (pRB and MMP-9), and these effects were exerted through direct binding with intact miR-9 binding site (Wt) but not its mutation sequence (Mut) (C). The symbol (#) indicates a significant increase from anti-NC. (PDF) [file pone.0055719.s004.pdf]

# Supplementary Figure S5

A

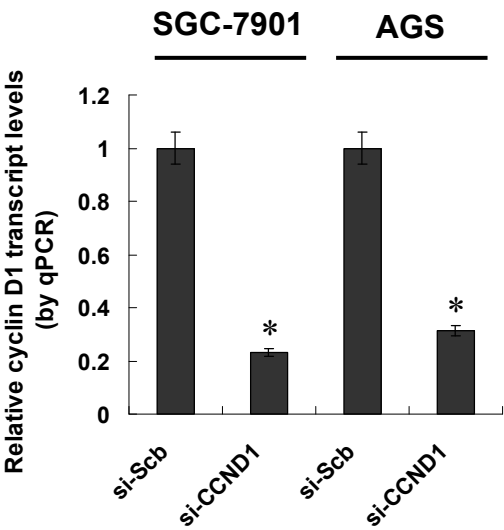

B

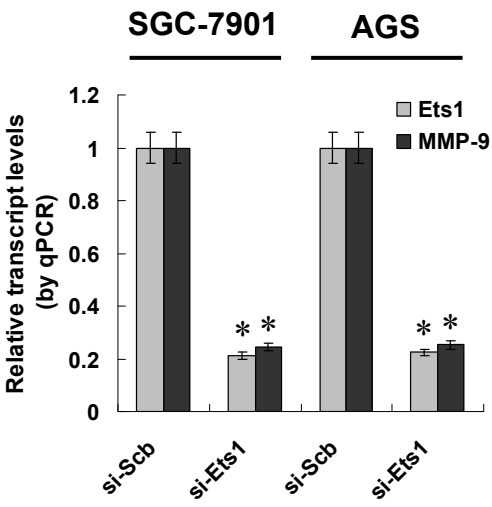

Supplement: Figure S5 — Knockdown of cyclin D1 and Ets1 in gastric cancer cells. Real-time quantiative RT-PCR indicated that transfection of si-CCND1 (100 nmol/L) and si-Ets1 (100 nmol/L) into gastric cancer SGC-7901 and AGS cells resulted in decreased transcript levels of cyclin D1 (A), Ets1 and its downstream gene MMP-9 (B) than those transfected with scramble siRNA (si-Scb, 100 nmol/L). The symbol (*) indicates a significant decrease from si-Scb. (PDF) [file pone.0055719.s005.pdf]
