# Supplementary material for: microRNA-9 Suppresses the Proliferation, Invasion and Metastasis of Gastric Cancer Cells through Targeting Cyclin D1 and Ets1
Source: PLoS One. 2013 Jan 31;8(1):e55719. doi: 10.1371/journal.pone.0055719 (PMC3561302; doi:10.1371/journal.pone.0055719)
Supplement: Table S1 — Cyclin D1, Ets1 and miR-9 expression in human gastric cancer tissues. (PDF) [file pone.0055719.s006.pdf]

Supplementary Table S1 Cyclin D1, Ets1 and miR-9 expression in human gastric cancer tissues

Clinicopathologic factor	Total	cyclin D1 expression		Ets1 expression		miR-9 expression	
	n (%)	n (%)	*P*-value	n (%)	*P*-value	mean (SEM)	*P*-value
**Age (years)**							
≤60	44 (51.2)	12 (27.3)	0.640	28 (63.6)	0.495	1.3541 (0.1583)	0.830
>60	42 (48.8)	14 (33.3)		30 (71.4)		1.3055 (0.1601)	
**Sex**							
Male	60 (69.8)	16 (26.7)	0.312	38 (63.3)	0.317	1.3387 (0.1239)	0.911
Female	26 (30.2)	10 (38.5)		20 (76.9)		1.3112 (0.2394)	
**Size (diameter)**							
≤6 cm	56 (65.1)	16 (28.6)	0.806	36 (64.3)	0.473	1.3261 (0.1289)	0.959
>6 cm	30 (34.9)	10 (33.3)		22 (73.3)		1.3383 (0.2154)	
**Laurén classification**							
Intestinal type	50 (58.1)	14 (28.0)	0.639	34 (68.0)	1.000	1.3730 (0.1301)	0.656
Diffuse type	36 (41.9)	12 (33.3)		24 (66.7)		1.2711 (0.1991)	
**Gastric wall invasion**							
T1/T2	22 (25.6)	0 (0.00)	<0.001	8 (36.4)	0.001	2.3127 (0.2062)	<0.001
T3/T4	64 (74.4)	26 (40.6)		50 (78.1)		0.9927 (0.1038)	
**Lymph node metastasis**							
Negative	22 (25.6)	0 (0.00)	<0.001	6 (27.3)	<0.001	1.9809 (0.2133)	<0.001
Positive	64 (74.4)	26 (40.6)		52 (81.3)		1.1067 (0.1200)	
**Distant metastasis**							
Negative	66 (76.7)	12 (18.2)	<0.001	38 (57.6)	<0.001	1.4705 (0.1201)	0.022
Positive	20 (23.3)	14 (70.0)		20 (100.0)		0.8680 (0.2522)	
**TNM stage**							
I/II	30 (34.9)	2 (6.7)	<0.001	14 (46.7)	0.004	1.7207 (0.1544)	0.010
III/IV	56 (65.1)	24 (42.9)		44 (78.6)		1.1213 (0.1439)	

Ets1, v-ets erythroblastosis virus E26 oncogene homolog 1; SEM, standard error of the mean
