# Supplementary material for: microRNA-9 Suppresses the Proliferation, Invasion and Metastasis of Gastric Cancer Cells through Targeting Cyclin D1 and Ets1
Source: PLoS One. 2013 Jan 31;8(1):e55719. doi: 10.1371/journal.pone.0055719 (PMC3561302; doi:10.1371/journal.pone.0055719)
Supplement: Table S2 — Primer sets used for RT-PCR and qPCR. (PDF) [file pone.0055719.s007.pdf]

Supplementary Table S2 Primer sets used for RT-PCR and qPCR

Primer set	Primers	Sequence	Product size (bp)	Application
cyclin D1	Forward	5'-TGAACTACCTGGACCGCT-3'	268	RT-PCR, qPCR
	Reverse	5'-GCCTCTGGCATTTTGGAG-3'		
Ets1	Forward	5'-TTCACTAAAGAACAGCAAC-3'	205	RT-PCR, qPCR
	Reverse	5'-TGTCCCCAACAAAGTCTG-3'		
MMP-9	Forward	5'-CAGAGATGCGTGGAGAGT-3'	220	RT-PCR, qPCR
	Reverse	5'-TCTTCCGAGTAGTTTTGG-3'		
β-actin	Forward	5'-ATCTACGAGGGGTATGCC-3'	227	RT-PCR, qPCR
	Reverse	5'-TAGCTCTTCTCCAGGGAG-3'		
miR-9	Forward	RiboBio		qPCR
	Reverse	RiboBio		
U6	Forward	RiboBio		qPCR
	Reverse	RiboBio		

Ets1, v-ets erythroblastosis virus E26 oncogene homolog 1; MMP-9, matrix metalloproteinase 9
