# Supplementary material for: microRNA-9 Suppresses the Proliferation, Invasion and Metastasis of Gastric Cancer Cells through Targeting Cyclin D1 and Ets1
Source: PLoS One. 2013 Jan 31;8(1):e55719. doi: 10.1371/journal.pone.0055719 (PMC3561302; doi:10.1371/journal.pone.0055719)
Supplement: Table S3 — Oligonucleotide sets used for constructs, miRNA inhibitor and small interfering RNAs. (PDF) [file pone.0055719.s008.pdf]

Supplementary Table S3 Oligonucleotide sets used for constructs, miRNA inhibitor and small interfering RNAs

Oligo Set	Sequences
Pre-miR-9	5'-TGCTGTCTTTGGTTATCTAGCTGTATGAGTTTTGGCCACT GACTGACTCATACAGAGATAACCAAAGA-3' (sense);<br>5'-CCTGTCTTTGGTTATCTCTGTATGAGTCAGTCAGTGGCCA AAACTCATAC AGCTAGATAACCAAAGAC-3' (antisense)
Negative control	5'-TGCTGAAATGTACTGCGCGTGGAGACGTTTTGGCCACTG ACTGACGTCTCCACGCAGTACATTT-3' (sense)<br>5'-CCTGAAATGTACTGCGTGGAGACGTCAGTCAGTGGCCAA AACGTCTCCACGCGCAGTACATTTC-3' (antisense)
pmiR-RB-cyclin D1 (wide type)	5'-CCGCTCGAGCCCCTGACAGTCCCTCCT-3' (sense);<br>5'-GAATGCGGCCGCTGGGGTTTTACCAGTTTTAT- 3' (antisense)
pmiR-RB-cyclin D1 (mutant)	5'-GTTGTGTGTATCGAGAGGCCTTAGGCTGGTGG -3' (sense);<br>5'-GGCCTCTCGATACACACAACATCCAGGACT- 3' (antisense)
pmiR-RB-Ets1 (wide type)	5'-CCGCTCGAGTGGCACTGAAGGGGCTGGGGAAA-3' (sense);<br>5'-GAATGCGGCCGCCTCTCCAGCAAAATGATG- 3' (antisense)
pmiR-RB-Ets1 (mutant)	5'-GTTTTTTCTCCTTTGTCCTTAGGCCAGAGAC-3' (sense)<br>5'-GGACAAAGGAGAAAAAACATCTGGCCAG-3' (antisense)
pcDNA3.1-cyclin D1	5'-CGCCCAAGCTTATGGAACACCAGCTCCTG-3' (sense)<br>5'-CTAGTCTAGATCAGATGTCCACGTCCCG-3' (antisense)
pcDNA3.1-Ets1	5'-CGCCCAAGCTTATGAGCTACTTTGTGGATTC-3' (sense)<br>5'-CTAGTCTAGATCACTCGTCGGCATCTGGCT-3' (antisense)
Anti-NC	RiboBio
Anti-miR-9	RiboBio
si-Scb	5'-GGAUUGGAAACGCAUAUAUtt-3' (sense);<br>5'-AUAUAUGCGUUUCCAAUCCtt-3' (antisene)
si-CCND1	5'-GUUCAUUUCCAAUCCGCCCtt-3' (sense);<br>5'-GGGCGGAUUGGAAAUGAACtt-3' (antisene)
si-Ets1	5'-AUAGAGAGCUACGAUAGUUtt-3' (sense);<br>5'-AACUAUCGUAGCUCUCUAUtt-3' (antisense)

Ets1, v-ets erythroblastosis virus E26 oncogene homolog 1; CCND1, cyclin D1; Anti-NC, negative control inhibitor; si-Scb, scramble siRNA
